# Electromechanical Behavior of Axially Continuous Graphene–Copper Wires

**DOI:** 10.1002/smsc.202500406

**Published:** 2025-10-14

**Authors:** Uschuas Dipta Das, Wonjune Choi, Hamid Safari, Jiali Yao, Wonmo Kang

**Affiliations:** ^1^ Mechaincal Engineering School for Engineering of Matter, Transport and Energy Arizona State University Tempe AZ 85281 USA; ^2^ Department of Materials Science and Engineering Dankook University Cheonan‐si Chungcheongnam‐do 31116 Republic of Korea; ^3^ Materials Science and Engineering, Fulton Schools of Engineering Arizona State University Tempe AZ 85287 USA

**Keywords:** electromechanical behavior, graphene–copper composites, graphene‐enhanced conductivity, microscale wires, microstructural characterization

## Abstract

Graphene–copper (Gr–Cu) composite conductors have demonstrated Gr‐enhanced electrical and thermal properties. However, the conductors’ coupled mechanical and electrical responses remain unexplored despite the importance of their mechanical flexibility and robustness. Here, the electromechanical behavior of a recently developed microscale Gr‐Cu composite, called axially continuous graphene–copper (ACGC) wire, has been investigated by developing and utilizing a customized tensile testing method. Experimental studies have shown that 80 μm‐diameter ACGC (hereafter ACGC80) wires exhibit 3.681% and 3.173% higher compared to as‐received and annealed Cu wires, respectively. More importantly, the Gr‐enhanced electrical performance of the ACGC80 has been observed even after significant plastic deformation under uniaxial tension. To be specific, the conductivity of ACGC80 is 3.139%, 3.144%, and 3.088% higher than that of annealed copper wire at 3, 6, and 9% strain, respectively. Analysis indicates that ACGC80 deforms by forming highly localized plastic deformation zones along its length. This result suggests that graphene in ACGC80 serves as an effective electron pathway even after applying a large strain because the pronounced damage to graphene is limited to only a small fraction of ACGC80. The ACGC80 conductor has great potential to advance emerging applications in flexible interconnects, wearable electronics, and high‐power transmission for microchips.

## Graphical abstract

This study investigates the electromechanical behavior of 80 μm‐diameter axially continuous graphene–copper (ACGC80) wires by developing a customized tensile testing method. Experimental studies have shown that ACGC80 wires exhibit higher electrical conductivity compared to as‐received and annealed Cu wires, and more importantly, the Gr‐enhanced electrical performance of the ACGC80 has been observed even after significant plastic deformation under uniaxial tension.

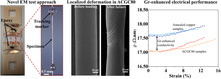

## 1 Introduction

Copper (Cu) is extensively used as a conductor for most electrical and electronic applications due to its excellent electrical and thermal properties, good flexibility and toughness, and relatively low cost. However, the ever‐increasing demands for better conductors, for example, from electric vehicles, high‐speed communication, and high‐performance electronics, are pushing copper conductors towards their intrinsic performance limits.^[^ ^1^ ^]^ Recently, graphene (Gr)—a 2D carbon allotrope with outstanding mechanical properties (Young's modulus of ≈1 TPa^[^ ^2^ ^]^ and failure strain of ≈20%^[^ ^3^ ^]^), thermal conductivity (≈5300 Wm^−1^ K^−1^^[^ ^4^ ^]^), and electron mobility (theoretically up to ≈20 m^2^ V^−1^ s^−1^^[^ ^5^ ^]^)—has garnered significant research interest for various applications, from flexible electronics to high‐frequency devices.^[^ ^6^, ^7^, ^8^, ^9^, ^10^, ^11^ ^]^ The unique characteristics of Gr have also made it a promising constituent for various composite systems with enhanced material properties.^[^ ^12^, ^13^, ^14^, ^15^, ^16^, ^17^, ^18^ ^]^ For example, graphene–copper (Gr–Cu) composites, integrating the advantages of both Gr and Cu, have presented promising research opportunities to surpass the electrical performance of traditional pure metal conductors for emerging high‐performance electronic and energy applications.^[^ ^19^, ^20^, ^21^ ^]^

Several techniques have been developed to synthesize Gr–Cu composites, including mechanical exfoliation, graphene oxide reduction, liquid‐phase mixing, electrodeposition, ball milling, and chemical vapor deposition (CVD).^[^ ^22^ ^]^ However, the continuity and uniformity of both the graphene network and copper matrix are crucial for the improvement of the electrical conductivity of Gr–Cu composites, making most synthesis methods unsuitable for achieving optimal electrical performance.^[^ ^23^ ^]^ For instance, while mechanical exfoliation is conceptually simple, it suffers from low exfoliation efficiency and increased defect density in Gr due to its mechanically violent processes (e.g., rolling and folding).^[^ ^24^, ^25^ ^]^ Likewise, reduced graphene oxide techniques can yield higher Gr volume fractions but often produce highly defective and non‐uniformly dispersed Gr.^[^ ^26^ ^]^ These techniques disperse Gr in metal matrices, but often damage Gr's structural integrity and produce nonuniform composites due to agglomeration and weak interfacial bonding.^[^ ^27^ ^]^ In contrast, CVD has emerged as a method for synthesizing high‐quality, large‐area Gr directly on a metal substrate, for example, on copper and nickel.^[^ ^28^, ^29^, ^30^, ^31^ ^]^ Copper's low carbon solubility promotes high‐temperature carbon source decomposition and self‐limiting growth of high‐quality single‐ or bilayer graphene, making direct growth on copper ideal for fabricating Gr–Cu composites without transfer steps.^[^ ^32^ ^]^ As a result, CVD‐grown Gr is increasingly considered to improve the electrical performance of Cu.

Several researchers have studied the electrical performance of CVD‐grown Gr on Cu thin foils or fine wires to maximize the available surface area for Gr growth. For example, Cao et al. achieved 117% IACS conductivity in graphene/copper composites produced via CVD‐graphene growth on 9 μm‐thick foils and then hot pressing of the multiple foils.^[^ ^33^ ^]^ The authors attributed the higher conductivity to the enhanced electron density in Gr through metal matrix doping while preserving graphene's high electron mobility. Similarly, Ding et al. reported a 7.83% improvement in the electrical conductivity of 20 μm‐thick copper foil by coating it with bilayer graphene and subsequently depositing a 100 nm‐thick copper nanofilm via vacuum evaporation, attributing the enhancement primarily to the presence of high‐quality, uniform, and low‐defect graphene.^[^ ^34^ ^]^ Another notable technical advance has been achieved by integrating axially bi‐continuous graphene and copper structures.^[^ ^35^, ^36^, ^37^ ^]^ These axially continuous graphene–copper (ACGC) wires, synthesized via CVD, have diameters in the range of 10‐80 μm and offer remarkably high electrical conductivity, enhanced charge‐carrying capacity, and better heat dissipation rates.^[^ ^35^ ^]^ Several previous studies mentioned above indicate that Gr–Cu composites, based on either thin Cu foils or fine Cu wires, exhibit superior electrical performance, making them particularly suitable for miniaturized electronic devices, wearable sensors, and high‐power transmission to microchips.^[^ ^35^, ^36^ ^]^ It is important to note that these emerging technologies often require both excellent electrical properties and mechanical deformability, as mechanical deformation can damage copper, graphene, and/or their interface, potentially leading to degradation of the Gr‐enhanced electrical conductivity.

In addition to conventional pure Cu,^[^ ^38^, ^39^, ^40^, ^41^ ^]^ mechanical properties of CVD‐grown Gr^[^ ^42^, ^43^, ^44^, ^45^ ^]^ have been well characterized. Cao et al. demonstrated through in situ SEM tensile tests that single‐crystalline monolayer graphene grown via CVD can achieve mechanical properties close to theoretical limits, even with edge defects.^[^ ^46^ ^]^ However, CVD‐grown Gr in metals is usually polycrystalline, with grain boundaries and defects that significantly compromise the strength and flexibility of Gr.^[^ ^47^, ^48^, ^49^ ^]^ For Gr–Cu composites, Na et al. reported the formation of cracks in polycrystalline Gr grown on Cu foils even under low tensile strain (≈0.5%), which is significantly lower than pristine graphene.^[^ ^47^ ^]^ It is worth noting that cracks, defects, and interface delamination in Gr–Cu conductors induced by mechanical deformation likely compromise their electrical conductivity. Hence, evaluating the ability of Gr–Cu composites to maintain superior electrical conductivity during mechanical deformation is imperative to predict and ensure long‐term reliability and applicability toward practical applications.

Despite the promising properties of Gr–Cu composites, their coupled mechanical and electrical properties are not fully understood, mainly due to a lack of an experimental approach. To address the current experimental challenges and knowledge gaps, this work investigates the electro‐mechanical coupling behavior of 80 μm‐diameter ACGC (hereafter ACGC80) composite wires by developing and utilizing a novel test approach for electromechanical characterization of fine wires. We first introduce and validate our new experimental method and then characterize the coupled electrical and mechanical behavior of ACGC80 wires compared to their annealed copper counterparts (hereafter Ann Cu80). The main finding of this study is that ACGC80 retains robust Gr‐enhanced electrical performance even under significant plastic deformation (e.g., until mechanical failure). This unique electromechanical behavior is explained by highly localized plastic deformation in ACGC80, characterized through careful postmortem analyses of both Gr and Cu. The results of this study are relevant to designing and optimizing graphene‐reinforced metal composites for future technologies, such as flexible electronics,microelectromechanical systems, and next‐generation energy storage devices.

## 2 Results and Discussions

### 2.1 Experimental Approach

Electromechanical characterization of microscale specimens poses technical challenges because their precise characterization requires high‐resolution measurements of both force and displacement, mainly due to small dimensions. Handling such delicate samples becomes nontrivial. In addition, robust electrical contacts to a specimen are required through probes without interfering with sensitive mechanical measurements during each uniaxial test. To address these challenges, an innovative electromechanical tester for microscale wire specimens was developed by integrating a 3D‐printed plastic sample holder with a conventional uniaxial tester. The sample holder is designed so that mechanical handling of a specimen is possible without applying any unwanted force to it, and a four‐probe configuration can be integrated for reliable and concurrent electrical characterization. More details on the developed electromechanical tester can be found in the Experimental Section.

**Figure 1:**
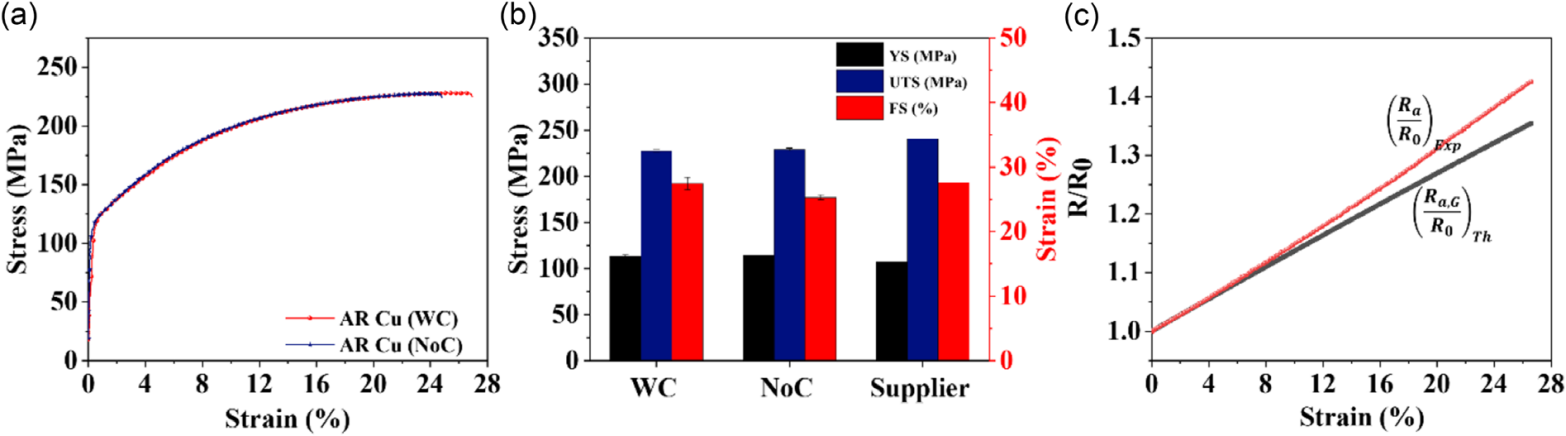
a) Representative stress–strain curves for 80 μm as‐received copper wires (hereafter AR Cu80) tested with applied current (WC) and without current (NoC), showing almost identical mechanical responses. b) Comparison of yield strength (YS), ultimate tensile strength (UTS), and failure strain (FS) for AR Cu80 under applied current (WC), without current (NoC), and supplier‐provided reference data. Error bars indicate standard deviations. c) Normalized resistance versus strain, demonstrating simultaneous electrical and mechanical response measurements during tensile deformation.

For electrical measurement, a small voltage of 0.7 mV was applied to a specimen to minimize possible Joule heating since it is well known that material properties depend on temperature (e.g., the temperature coefficient of resistance of copper is $3.9\times 10^{-3}$ /K^[^ ^50^ ^]^). Moreover, Ross et al. reported reductions in flow stress and elastic modulus with increasing current density during tensile deformation of copper.^[^ ^51^ ^]^ Despite the use of low voltage, the corresponding current density was still relatively high (≈4 A mm^−2^) due to the small cross‐section of a specimen, and therefore, we conducted both numerical simulation and experiments to confirm that the use of such a small input voltage eliminated the temperature effect. Our finite element analysis (FEA) studies using COMSOL predicted constant temperature in a copper specimen at 4 A mm^−2^ (see details in the Supporting Information, including Figure S1, S2). For experimental confirmation, **Figure** **1**a shows the stress–strain curves of two 80 μm‐diameter as‐received copper wires (or AR Cu80), one with and the other one without applying 0.7 mV. It is obvious that the two specimens exhibited almost identical mechanical behavior, matching the conclusion of our FEA study. For quantitative comparison, Figure 1b summarizes the yield strength (YS, black bar), ultimate tensile strength (UTS, blue bar), and failure strain (FS, red bar) of specimens with and without applied current. The average yield stress and ultimate tensile stress for specimens without current application were 113.79 and 229.26 MPa, respectively. Comparatively, the YS and UTS for specimens tested under a 4 A mm^−2^ current density were 113.60 and 227.48 MPa. Note that these values match well with the data provided by a vendor (indicated as “Supplier” in Figure 1b).

For electrical measurements, Figure 1c shows experimentally measured resistance (*R*) of AR Cu80 as a function of strain (*ε*) (see a red curve). Note that *R* is normalized by its initial value ($R_{0}=37.201\text{ mΩ}$). It is worth noting that the experimentally measured $R_{0}$ matches well with an expected value based on the geometry of the wire and the well‐known electrical conductivity of copper. For further validation, consider a copper wire specimen with its initial length $L_{0}$ and radius $r_{0}$. When uniaxial tension is applied, a specimen is stretched and, thus, the cross‐sectional area is reduced. During deformation, the volume of a wire remains constant (i.e., $V_{0}=V_{a}$ where $V_{0}$ and $V_{a}$ are the wire volume before and after applying the uniaxial load $F_{a}$). Assuming uniform tensile deformation, it is obvious to show that $\pi r_{0}^{2}L_{0}=\pi r_{a}^{2}L_{a}$ or $r_{0}^{2}/r_{a}^{2}=L_{a}/L_{0}$ where $L_{a}$ and $r_{a}$ are the length and radius under $F_{a}$ (see the notations in Figure S3 in the Supporting Information). Now the resistivity ($\rho_{a}=\rho_{0}+\Delta \rho$) of a wire under $F_{a}$ can be written as

(1) $$\rho_{a}=\frac{R_{a}A_{a}}{L_{a}}=\frac{R_{a}\pi r_{0}^{2}L_{0}}{L_{a}^{2}}$$

where $R_{a}$ is the current resistance of the wire. It is important to note that the normalized resistance (${\bar{R}}_{a}=R_{a}/R_{0}$) can be written as

(2) $${\bar{R}}_{a}=\frac{R_{a,G}\;}{R_{0}}+\frac{R_{a,P}\;}{R_{0}}={\bar{R}}_{a,G}+{\bar{R}}_{a,P}={\left(\frac{L_{a}}{L_{0}}\right)}^{2}+\frac{\Delta \rho }{\rho_{0}}{\left(\frac{L_{a}}{L_{0}}\right)}^{2}$$

where $R_{a,G}$ and $R_{a,P}$ capture the resistance of a wire associated with geometrical and intrinsic property changes, respectively. Equation (2) can be rearranged as below to determine Δρ

(3) $$\Delta \rho =\rho_{0}\left(\frac{R_{a}}{R_{0}}{\left(\frac{L_{0}}{L_{a}}\right)}^{2}-1\right)$$

In the following sections, Equation (3) is used to analyze the intrinsic resistivity change (*Δρ*) of the specimen during the electromechanical test.

Figure 1c shows the theoretical prediction of ${\bar{R}}_{a,G}$ (see a black curve) based on Equation (2). Note that the two curves based on experiments and Equation (2) overlap until ε=4.44%, to which the change in resistance is fully captured by dimensional changes due to the applied load. For ε>4.44%, ${\bar{R}}_{a,P}={\left({\bar{R}}_{a}\right)}_{\text{Exp}}-{\bar{R}}_{a,G}>0$, indicating that intrinsic resistivity increases are likely associated with large plastic deformation. For example, considerable resistivity increases (4–7%) in pure copper were reported after large plastic deformation because of additional electron scattering at dislocations and grain boundaries.^[^ ^52^ ^]^

In this section, the development of reliable experimental protocols for the accurate characterization of coupled electrical and mechanical properties of small‐scale specimens has been discussed. Moreover, Table S2, Supporting Information, shows that the resistivity of AR Cu80 specimens measured by two different methods (i.e., the four‐point measurement in Figure S4 in the Supporting Information and the electromechanical tester) matches quite well. Figure S5 (Supporting Information) further illustrates the stress–strain plot and resistivity–strain plot for AR Cu80 specimens.

### 2.2 Characterization of Synthesized Axially Continuous Graphene–Copper Wires

The objective of this study was to investigate the Gr‐enhanced electromechanical responses of 80 μm‐diameter ACGC (hereafter ACGC80) composite wires, compared to their annealed counterpart, by quantitatively measuring coupled electrical and mechanical properties utilizing the developed electromechanical testing method validated by using AR Cu80 wires. The ACGC80 composite wires were synthesized by using the CVD method at 960 °C (see Experimental Section for detailed synthesis process). For the analysis of ACGC80 using Raman spectrum, it is worth noting that graphene–copper composites show three distinctive peaks at ≈1350, ≈1580, and ≈2690 cm^−1^, corresponding to the D, G, and 2D‐peaks, respectively.^[^ ^53^ ^]^ The intensity ratio of 2D to G peaks (or *I*_2D_/*I*_G_) and full width at half maximum (FWHM) of the 2D peak of Raman spectra can be used to estimate the number of graphene layers. More specifically, the intensity ratio of *I*_2D_/*I*_G_ is usually greater than 2 and the FWHM of the 2D peak is less than 45 cm^−1^ for monolayer graphene. In contrast, the *I*_2D_/*I*_G_ ratio of bilayer graphene is typically between 1 and 2, and the corresponding FWHM of 2D peak is in the range of 45‐60 cm^−1^.^[^ ^35^, ^54^ ^]^

**Figure 2:**
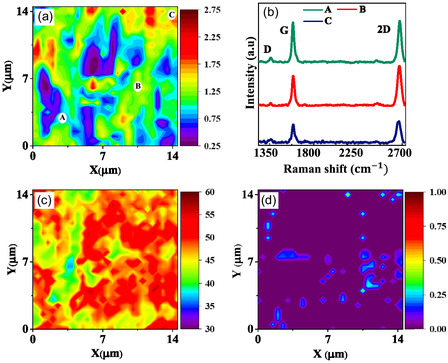
Raman mapping of synthesized graphene‐coated copper composite (ACGC80) wire: a) the intensity ratio of the 2D and G peaks (*I*_2D_/*I*_G_); b) the representative Raman spectra corresponding to locations A, B, and C indicated in (a); c) the FWHM of the 2D peak; and d) the intensity ratio of the D and G peaks (*I*_D_/*I*_G_).

**Figure** **2** presents the Raman mapping of ACGC80, performed over a 14.5 × 14.5 μm area with a 0.5 μm step size. Figure 2a shows the mapping of the *I*_2D_/*I*_G_ ratio with the average value of 1.04, indicating that bilayer graphene is dominant. Figure 2b shows Raman spectra of three different locations (A, B, and C in Figure 2a) with the D, G, and 2D peak positions around 1350, 1590, and 2680 cm^−1^. Additionally, Figure 2c,d demonstrates the FWHM of 2D peaks and the *I*_D_/*I*_G_ ratio, respectively. Most 2D peak FWHM falls between 40 and 55 cm^−1^, suggesting bilayer graphene coverage is dominant. D peaks with minimal intensity are observed near ≈1350 cm^−1^ in only a few locations, suggesting a continuous, high‐quality graphene layer with minimal disorder or defects in the composite wires. Additional details on Raman analysis can be found in Figure S6 (Supporting Information).

**Figure 3:**
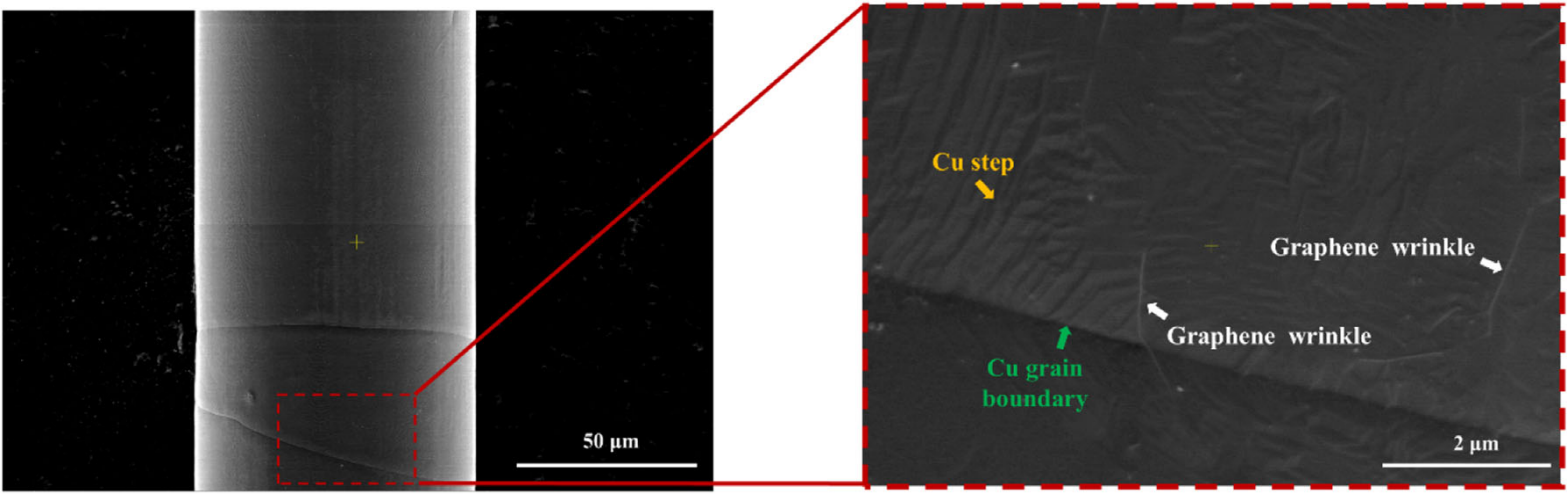
SEM images of the same 80 μm‐diameter ACGC wire. The low‐magnification image (left) shows the overall wire morphology, while the high‐magnification inset (right) reveals microstructural features such as Cu grain boundaries, graphene wrinkles, and Cu steps.

**Figure** **3** shows SEM images of the ACGC80 at different magnifications. The low‐magnification image (left) in Figure 3 illustrates the surface morphology, whereas the high‐magnification inset (right) shows detailed features such as graphene wrinkles, copper steps, and copper grain boundaries (white, yellow, and green arrows, respectively). The low‐magnification view highlights the formation of large grains in Cu (hereafter bamboo‐like microstructure; see Figure S7 in the Supporting Information for details) due to thermal grain growth during CVD. In the magnified view, a distinct Cu grain boundary can be observed, with a graphene wrinkle extending across it. Immediately after CVD graphene growth at 960 °C, the furnace was cooled down to room temperature, which causes thermally induced residual stress at the Gr–Cu interface because of the mismatch in their thermal expansion coefficients ($\alpha_{\text{copper}}=18\times 10^{-6}{\text{ K}}^{-1}\;$ and $\alpha_{\text{graphene}}=-7\times 10^{-6}{\text{ K}}^{-1}$). It is well known that such thermal stress results in the formation of graphene wrinkles and copper steps.^[^ ^55^, ^56^ ^]^ The degree of surface wrinkles depends on the cooling rate, the crystallographic orientation of copper, and the sublimation of copper at high temperatures.^[^ ^56^, ^57^ ^]^

### 2.3 Electromechanical Behavior of ACGC Wires

**Figure 4:**
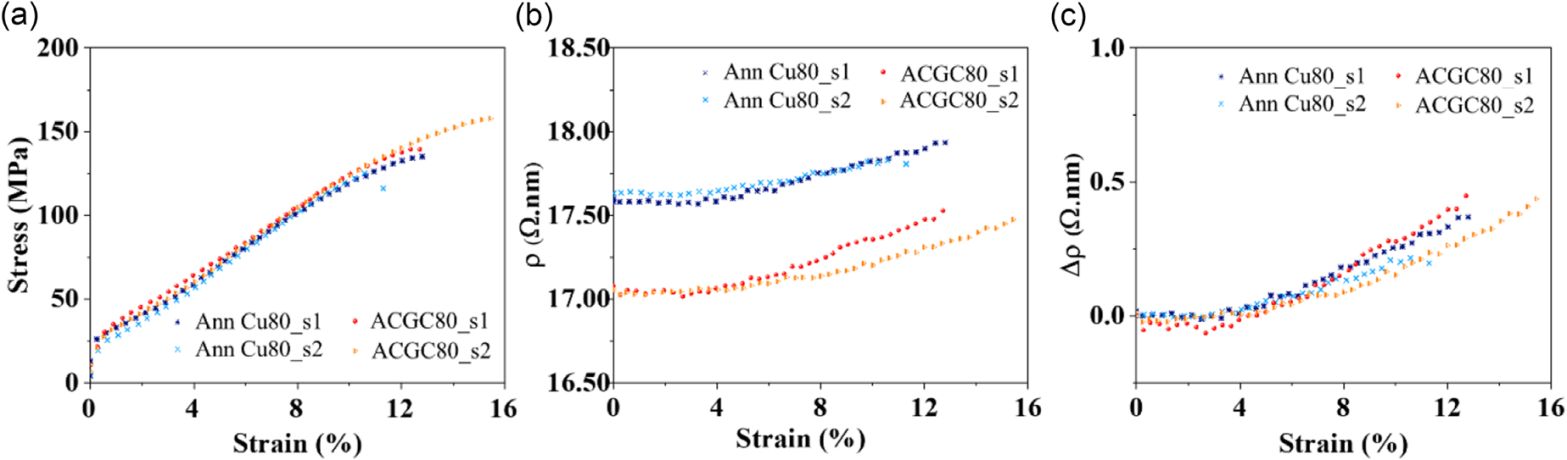
Electromechanical characterization of annealed (Ann Cu80) and graphene‐coated copper (ACGC80) wire specimens: a) stress–strain curves showing mechanical response under tensile loading; b) absolute resistivity values as a function of strain, highlighting the enhanced electrical conductivity of ACGC80 compared to Ann Cu80; and c) corresponding relative change in resistivity (Δ*ρ*) as a function of applied strain.

Using our custom‐built tester, the electromechanical behaviors of ACGC80 composite wires and their annealed counterparts (Ann Cu80) were analyzed using the same approach employed for AR Cu80. Since both ACGC80 and Ann Cu80 samples experienced identical thermal histories, a direct comparison of their electromechanical responses is meaningful. **Figure** **4**a,b shows stress and resistivity as a function of applied strain for Ann Cu80 and ACGC80 wires, respectively. Moreover, Figure 4c shows how the resistivity (Δ*ρ*) changes with strain plot for Ann and ACGC specimens. Despite having quite comparable flow stress and yield strength, ACGC80 wires exhibit slightly higher ductility (16.74%) and UTS (14.83%) on average than their annealed counterparts. This is an interesting observation because the volume fraction of graphene in the 80 μm‐diameter ACGC is still very limited. This observation can be explained by two mechanisms. First, the continuous graphene with outstanding mechanical properties (e.g., Young's modulus: 1 TPa and ultimate tensile strength: 100–130 GPa) can directly share mechanical loading with copper wire.^[^ ^17^, ^46^ ^]^ Furthermore, the surface of a copper wire is passivated by strong graphene, and therefore, dislocation escape to the free surface can be limited as observed in graphene‐coated nickel wires.^[^ ^58^ ^]^ It is worth discussing that the yield strength and flow stress of AR Cu80 (see Figure 1a) are significantly higher compared to both the Ann Cu80 and ACGC80 due to grain growth during thermal processes. As shown in the EBSD images in Figure S8a–c of the Supporting Information, the AR Cu80 exhibits a fine, equiaxed grain structure, whereas both ACGC80 and Ann Cu80 undergo significant grain coarsening, with grain sizes increasing from a few microns to tens of microns. Therefore, the observed softening (lower yield and flow stresses) in the Ann Cu80 and ACGC80 samples can be explained by the Hall–Petch relationship.^[^ ^58^, ^59^, ^60^ ^]^ To further quantify grain size, mechanical polishing was performed followed by chemical etching (using 0.5M FeCl_3_ etchant for 7 s) on both Ann Cu80 and ACGC80 specimens. The resulting optical microscopy images (Figure S8d,e, Supporting Information) revealed elongated grains spanning across the wire diameter, aligned parallel to the loading direction. The average grain size measured in the axial direction is 46.35 and 47.78 μm, respectively, for Ann Cu80 and ACGC80, indicating no significant microstructural difference.

Figure 4b shows that ACGC80 achieves 3.173% lower resistivity, compared to their annealed counterparts, at ε=0. The higher electrical conductivity (101.03% IACS) of ACGC is mainly due to axially bi‐continuous graphene and copper structures. The high‐quality continuous graphene forms a direct electron pathway in parallel to the copper wire and directly contributes to the overall electrical properties of ACGC80.^[^ ^35^ ^]^ More interestingly, experimental observations revealed that the Gr‐enhanced electrical conductivity is not sensitive to *ε*. Specifically, ACGC80 retained superior conductivity relative to their annealed counterparts, even after substantial plastic deformation, e.g., 3.043%, 3.048%, and 2.995% lower resistivity of ACGC80 (equivalent to 3.139%, 3.144%, and 3.088% higher conductivity) at *ε* = 3%, 6%, and 9% (see Figure 4b). This mechanically robust conductivity over a broad range of material deformation (i.e., graphene‐enhanced electrical conductivity of ACGC80 persisted even under significant mechanical deformation) is crucial toward many practical applications of ACGC, which is counterintuitive because large plastic deformation could damage Gr in ACGC and diminish Gr‐enhanced electrical conductivity. For example, the conventional Cu–Gr composites, based on discrete graphene flakes dispersed in a Cu matrix, commonly suffer from lower conductivity because discontinuous Gr‐to‐Gr or Gr‐to‐Cu interfaces cause additional electron scattering.^[^ ^23^ ^]^ To quantify changes in resistivity associated with damage in Gr, Equation (3) was utilized to calculate Δ*ρ* of ACGC80 and Ann Cu80 as shown in Figure 4c. ACGC80 and Ann Cu80 show very similar trends in Δ*ρ*, indicating that Gr‐enhanced electrical conductivity in ACGC is insensitive to applied mechanical strain. To reveal the underlying mechanisms of this mechanically robust electrical conductivity of ACGC80, we performed postmortem analysis in the following section.

### 2.4 Mechanisms for the Gr‐Enhanced Mechanical and Electrical Responses

**Figure 5:**
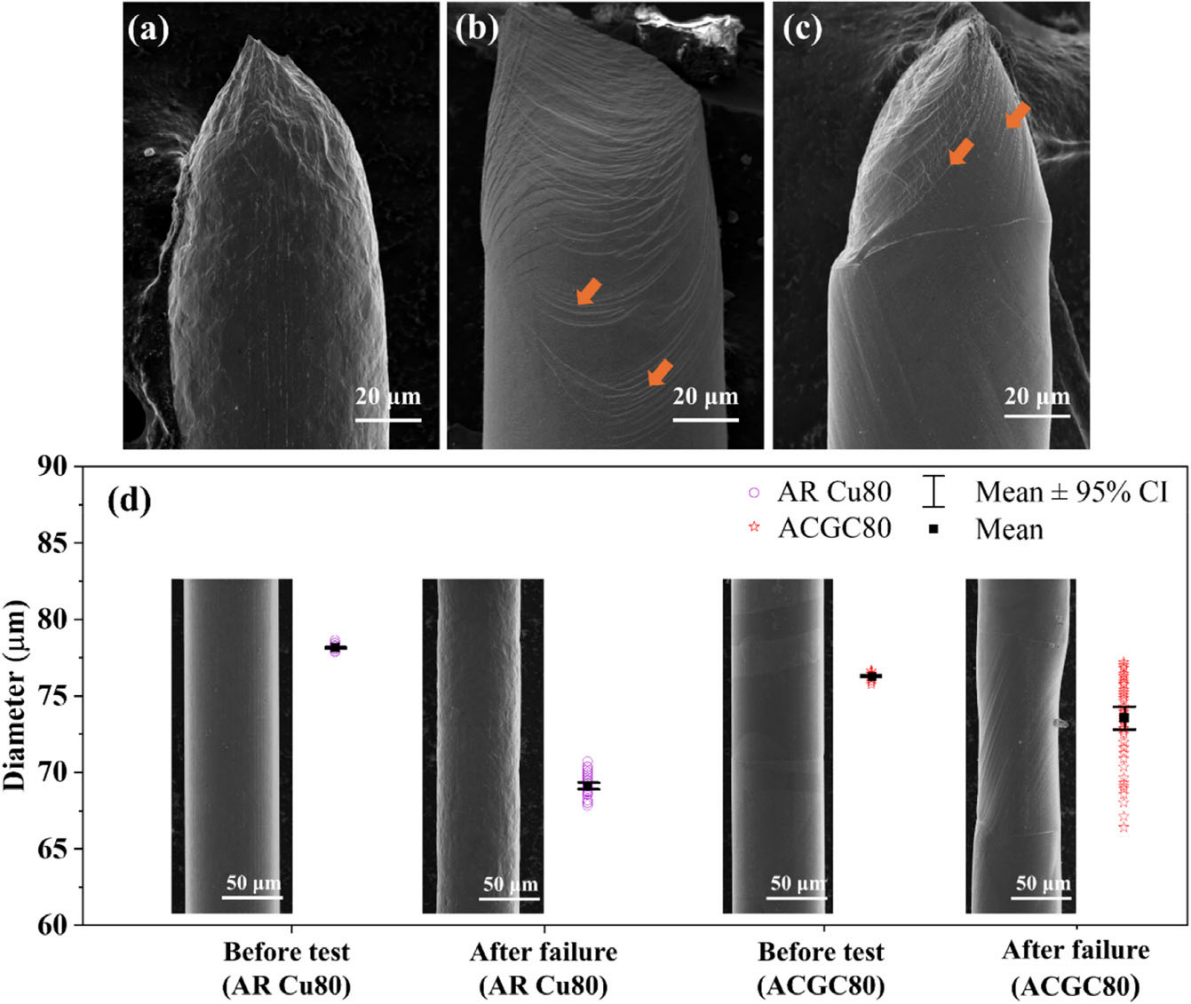
Fracture morphology of three types of wires: a) AR Cu80, b) Ann Cu80, and c) ACGC80. Orange arrows highlight prominent slip bands observed on the fracture surfaces of Ann Cu80 and ACGC80 specimens. d) Representative SEM micrographs showing wire morphology before and after tensile failure for AR Cu80 and ACGC80, along with corresponding diameter measurements at multiple locations. Both SEM images and measured diameter illustrate significant nonuniform deformation behavior in ACGC80 wires compared to polycrystalline AR Cu80.

**Figure** **5**a presents the fracture morphology of AR Cu80 with a polycrystalline microstructure (see EBSD in Figure S8a, Supporting Information). These grain boundaries restrict dislocation movement, acting as effective barriers due to crystallographic mismatch and misorientation, and as a result, no slip bands are observed near the fracture tip. In contrast, Ann Cu80 and ACGC80 in Figure 5b,c exhibit a bamboo‐like microstructure (see Figure S8b,c, Supporting Information) with elongated grains aligned parallel to the loading direction (i.e., one grain within the cross section; see Figure S7, Supporting Information). During tensile tests, dislocations within the preferred orientation grains can readily migrate to the free surface and form pronounced slip bands, as observed in the Ann Cu80 and ACGC80 composite wires in Figure 5b,c.

Figure 5d presents representative SEM micrographs of AR Cu80 and ACGC80 before and after tensile testing, including the measured diameters along each wire. It is worth noting that the diameters were measured about 1 mm away (or 12.5 times of diameter away) from the fractured tips to eliminate the effect of the necked area. The average diameter of AR Cu80 wire was measured as 78.17 ± 0.06 μm (95% confidence interval) prior to mechanical loading. AR Cu80 wires, even after failure at about 28% strain, maintained a relatively uniform cross‐sectional geometry with an average diameter of 69.14 ± 0.21 μm. In contrast, ACGC80 wire had a smaller initial diameter (76.3 ± 0.07 μm) than AR Cu80 due to the evaporation of copper during thermal annealing at a high‐temperature and low‐pressure environment used for graphene growth.^[^ ^35^ ^]^ After the sample failure of ACGC80, its average diameter was reduced to 73.23 ± 0.77 μm, about 6% larger than the failed AR Cu80. This observation can be simply explained by the different failure strains (i.e., ACGC80 failed at about 15% lower strain than AR Cu80). A more interesting observation is that ACGC80 wires exhibit inhomogeneous cross‐sectional reduction. For example, its diameter along gauge length varies in a broad range from 67 to 76.8 μm as summarized in Figure 5d. This nonuniform deformation behavior stems from the bamboo‐like microstructure of ACGC80 (also observed in annealed copper), where grains with a preferred Schmid factor yield first and experience more pronounced cross‐section reduction. Furthermore, this plastic deformation zone forms away from the fractured tip, strongly suggesting the formation of multiple localized plastic deformation zones along the gauge length. Figure S9 (Supporting Information) includes SEM images and diameter measurements of Ann Cu80 before and after the test, confirming that Ann Cu80 also exhibits localized deformation similar to ACGC80.

**Figure 6:**
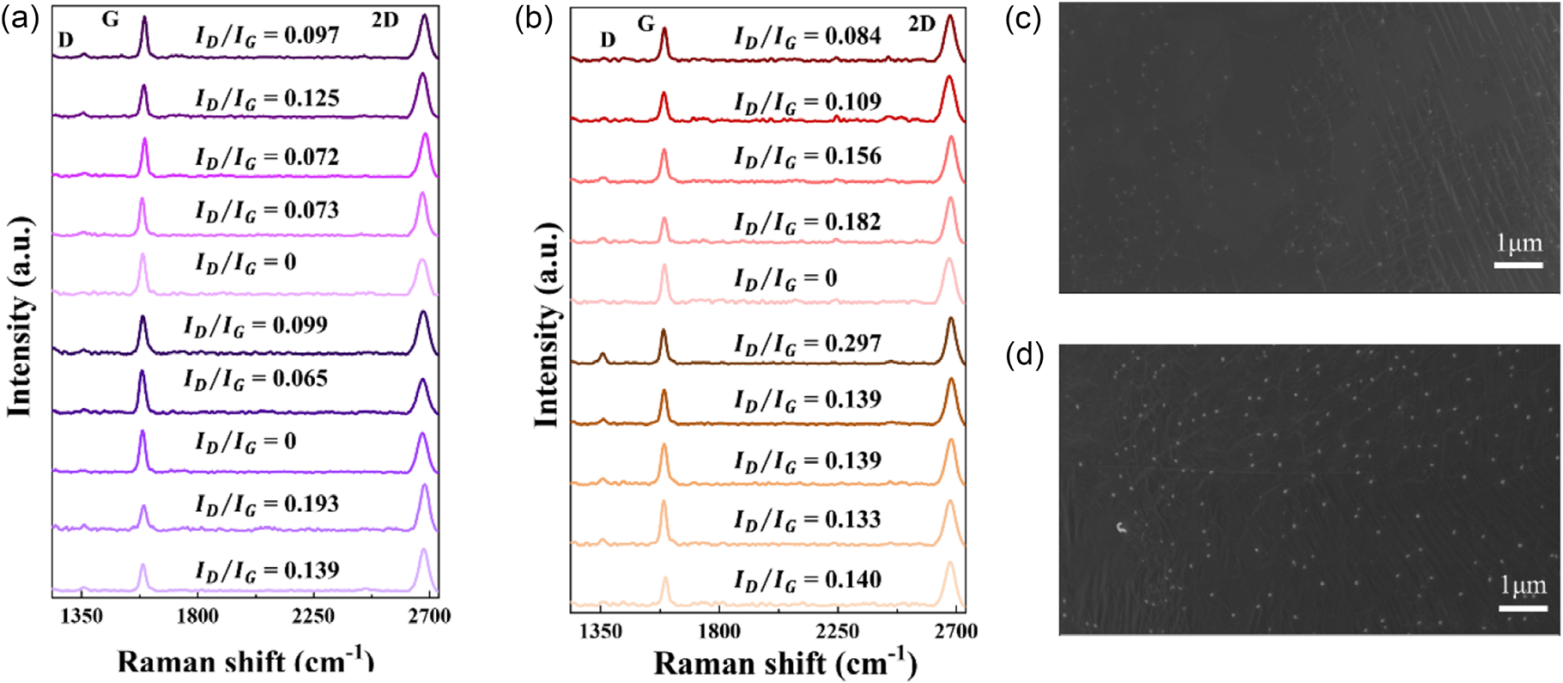
Raman and SEM analysis of ACGC80 wires before and after tensile testing. a,b) The D peak intensity and average *I*_D_/*I*_G_ ratio increased from 0.086 ± 0.043 (95% confidence interval) to 0.138 ± 0.054, illustrating minimal changes in D‐peak intensity and *I*_D_/*I*_G_ ratios. c,d) High‐magnification (10 000×) SEM images further confirm the absence of visible cracks in the graphene layer, even after failure: (c) SEM image of pristine ACGC80 wire and (d) SEM image of ACGC80 wire after tensile test.

To support localized plastic deformation, postfailure ACGC80 samples were analyzed via Raman spectroscopy with an emphasis on D (1350 cm^−1^), D′ (1620 cm^−1^), and D + G (2940 cm^−1^) because they are indicators of defects in graphene.^[^ ^53^ ^]^ Ten Raman spectra were randomly collected along the axial direction of the same ACGC80 wire before and after testing, as shown in **Figure** **6**a,b, respectively. By comparing these, the *I*_D_/*I*_G_ ratio increases from 0.086 ± 0.043 to 0.138 ± 0.054. This change is relatively small compared to the results from excessively damaged graphene, for example, by mechanical rolling in Figure S10 in the Supporting Information. We will revisit this below using graphene‐coated copper foils as a model for more quantitative comparison. Moreover, Figure 6c,d, illustrates high‐magnification (10 000×) SEM images of ACGC80 before loading and after failure, further confirming the absence of visible cracks in the graphene layer, even after failure.

**Figure 7:**
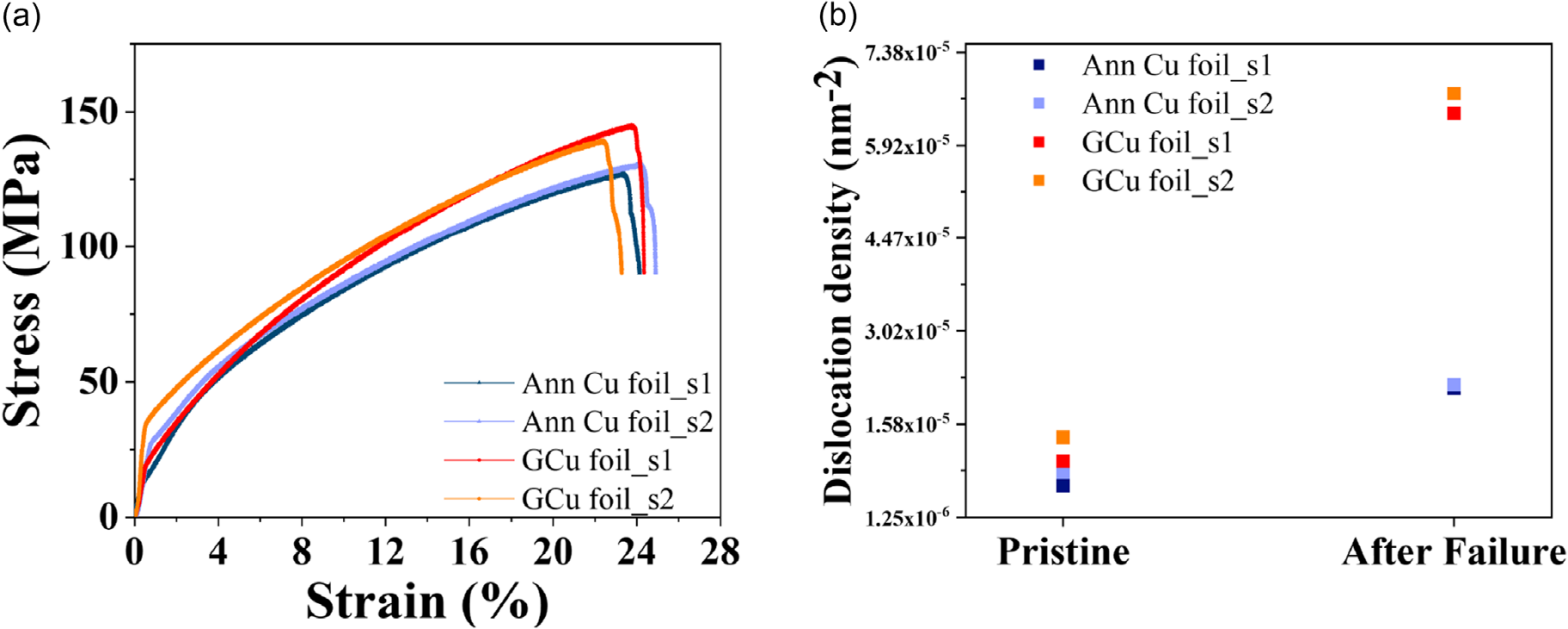
XRD analysis of graphene‐coated copper foil before and after tensile testing: a) Stress–strain plot for Ann Cu and GCu foil specimens. b) XRD results revealed a higher initial as well as larger relative increase in dislocation density (after deformation) in GCu compared to Ann Cu foil.

To explore the Gr‐enhanced mechanical properties, graphene‐coated copper (GCu) foils were prepared and tested because GCu, while its thickness (85 μm) is similar to ACGC80, still has multiple grains in its cross section (see Figure S11, Supporting Information) and, therefore, preserves the characteristics of polycrystalline Cu (e.g., the failure strain of GCu is about 24%, similar to AR Cu80). These GCu samples were synthesized by following the same process of ACGC explained in the Experimental Section. More details on GCu can be found in Figure S11 in the Supporting Information. **Figure** **7**a shows stress–strain responses of GCu foils under uniaxial tensile load. GCu samples exhibit higher flow stress compared to annealed copper (Ann Cu), for example, 10.38% higher at 15% strain. This increase is likely associated with the surface passivation of copper by strong graphene. In other words, graphene prevents dislocation escape to the free surface and, therefore, causes dislocation accumulation at the graphene–copper interface. Dislocation density measurements via XRD in Figure 7b also support this mechanism. Prior to applying any mechanical load, as‐prepared GCu and Ann Cu foil samples (indicated as “Pristine”) have comparable dislocation density. However, GCu foils show about three times higher dislocation density (1.19 × 10^−5^ to 6.58 × 10^−5^ nm^−2^) compared to Ann Cu (7.22 × 10^−6^ to 2.16 × 10^−5^ nm^−2^) after failure. Note that this observation agrees with graphene‐coated nickel wires.^[^ ^58^ ^]^ For instance, bilayer graphene‐coated ACGN wires (100 μm in diameter) exhibited 81.9%, 70.1%, and 21% higher yield strength, ultimate strength, and ductility, respectively, compared to annealed counterparts. Interestingly, Gr‐enhanced mechanical properties in nickel are notably higher than copper because the copper–graphene interface is approximately seven times weaker than the nickel–graphene interface (6.775 ± 0.556 J m^−2^).^[^ ^61^ ^]^

**Figure 8:**
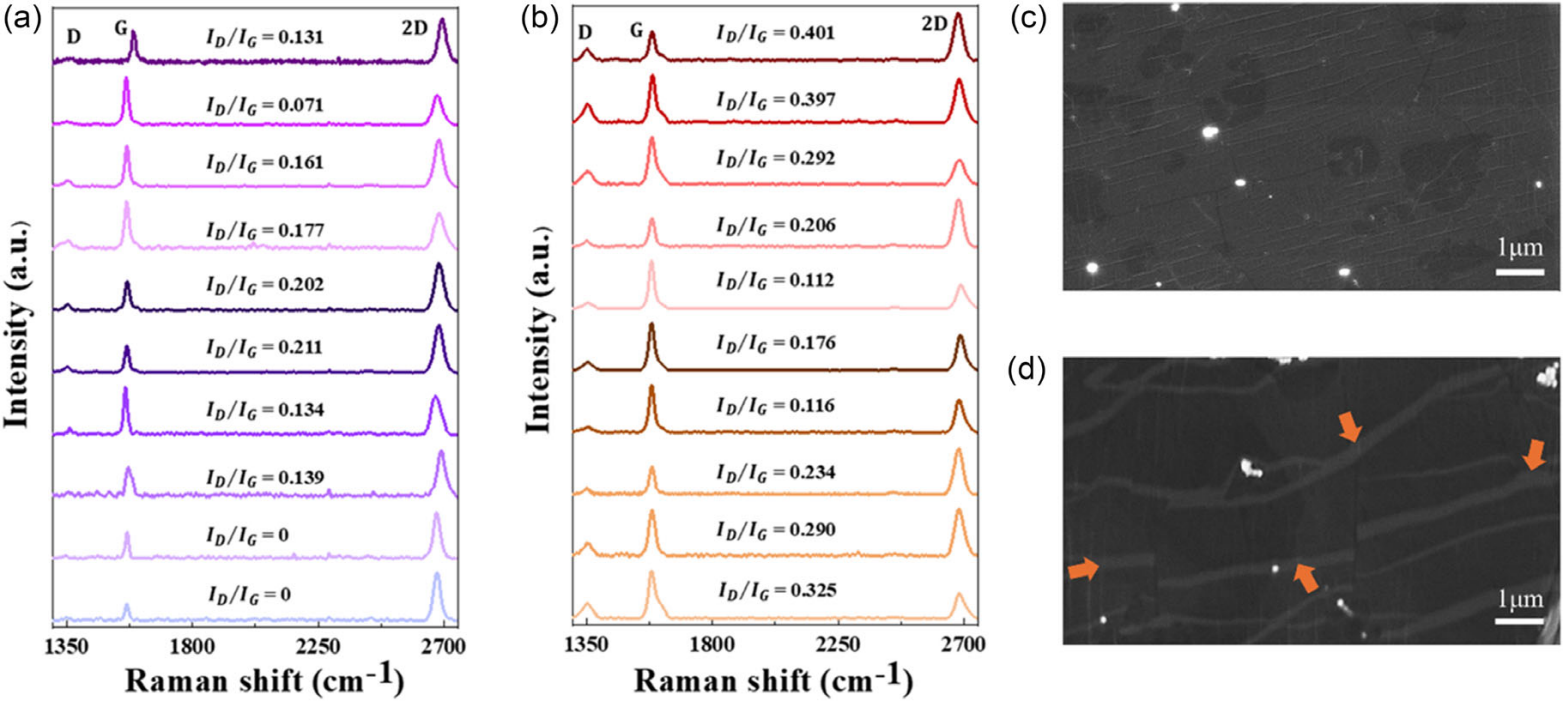
Raman spectroscopy and SEM analysis of graphene‐coated copper foil before and after tensile testing. a,b) The D peak intensity and average *I*_D_/*I*_G_ ratio increased from 0.123 to 0.255, indicating significant defect generation. c) SEM image of pristine graphene‐coated copper (GCu) foil. d) SEM image of GCu foil after failure, showing extensive crack formation (orange arrows were used to indicate the formed crack) throughout the surface.

To further characterize crack formation in graphene, the pristine and deformed GCu foil were analyzed via Raman spectroscopy and SEM. In contrast to ACGC80 wires, the GCu foil subjected to uniaxial tensile load has shown a more pronounced increase in *I*_D_/*I*_G_ ratio, increased from 0.123 ± 0.055 to 0.255 ± 0.075, indicating higher defect density compared to deformed ACGC80, mainly due to the excessive crack formation in the graphene (see **Figure** **8**a,b). This is consistent with the SEM images of the GCu foil before and after the tensile test (see Figure 8c,d) where obvious cracks can be observed in the latter. These conclusions are clearly different from ACGC80, supporting that the localized plastic deformation of ACGC80 results in localized damage to graphene even after severe plastic deformation. This suggests that the graphene network in ACGC80 (Figure 6c,d) maintains its overall continuity over a wide range of deformation and, therefore, ACGC80 achieves mechanically robust electrical conductivity until its failure strain.

In summary, the mechanically robust electrical conductivity of ACGC80 can be explained by the postmortem analysis. ACGC80 deforms in a highly localized manner, and therefore, the corresponding damage to Gr becomes highly heterogeneous as our Raman and SEM analyses suggest. We conclude that ACGC retains its enhanced electrical conductivity under large tensile deformation mainly because damage to Gr is limited to a small fraction of ACGC80. Note that thermally induced residual stress in ACGC80 may also contribute to the robustness of ACGC80 within relatively small strain. After CVD at 960 °C, ACGC80 rapidly cools down to room temperature. During this cooling, Cu contracts by about 1.68% due to its positive thermal expansion coefficient (18 × 10^−6^ K^−1^) while Gr expands by about 0.23% due to its negative thermal expansion coefficient (−2.5 × 10^−6^ K^−1^ over this temperature range^[^ ^62^ ^]^). This thermal expansion mismatch induces compressive strain in the graphene layer (about 1.92%), and as a result, it can be argued that Gr in ACGC80 can accommodate relatively small strain by unfolding of thermally induced wrinkles and ripples.^[^ ^63^ ^]^

## 3 Conclusion

Interest in graphene–copper composite for electrical applications is ever growing because of its exceptional properties enhanced by graphene. Despite the significant potential for graphene–copper composite wires (ACGC) in high‐power transmission and miniaturized electronic applications, particularly due to their excellent thermoelectrical performance and stability at elevated temperatures, their electrical behavior under stress remained unexplored. This study addressed the gap by investigating the electromechanical coupling behavior of ACGC80 wires.

ACGC80 wires exhibit Gr‐enhanced mechanical and electrical properties compared to their Ann Cu80 counterparts: 16.74% higher ductility, 14.83% ultimate tensile strength, and 3.173% higher electrical conductivity (101.03% IACS). The continuous graphene in ACGC80 acts as 1) a surface passivation layer that prevents dislocation annihilation to the free surface and 2) an effective electron transfer pathway. Remarkably, ACGC80 wires retain Gr‐enhanced electrical performance even after significant plastic deformation.

In conclusion, ACGC wires exhibit mechanically robust electrical conductivity across a wide deformation range, widely broadening the potential applications of ACGC, including energy‐efficient EV motors, wire bonding for microchips, and wearable devices. The fundamental understanding of the electromechanical responses of ACGC80 is relevant to innovative design and synthesis of high‐performance graphene–metal technologies, such as flexible electronics, microelectromechanical systems (MEMS), and next‐generation energy storage devices.

## 4 Experimental Section

4.1

4.1.1

##### Innovative Electromechanical Tester for Fine Wire Samples

**Figure 9:**
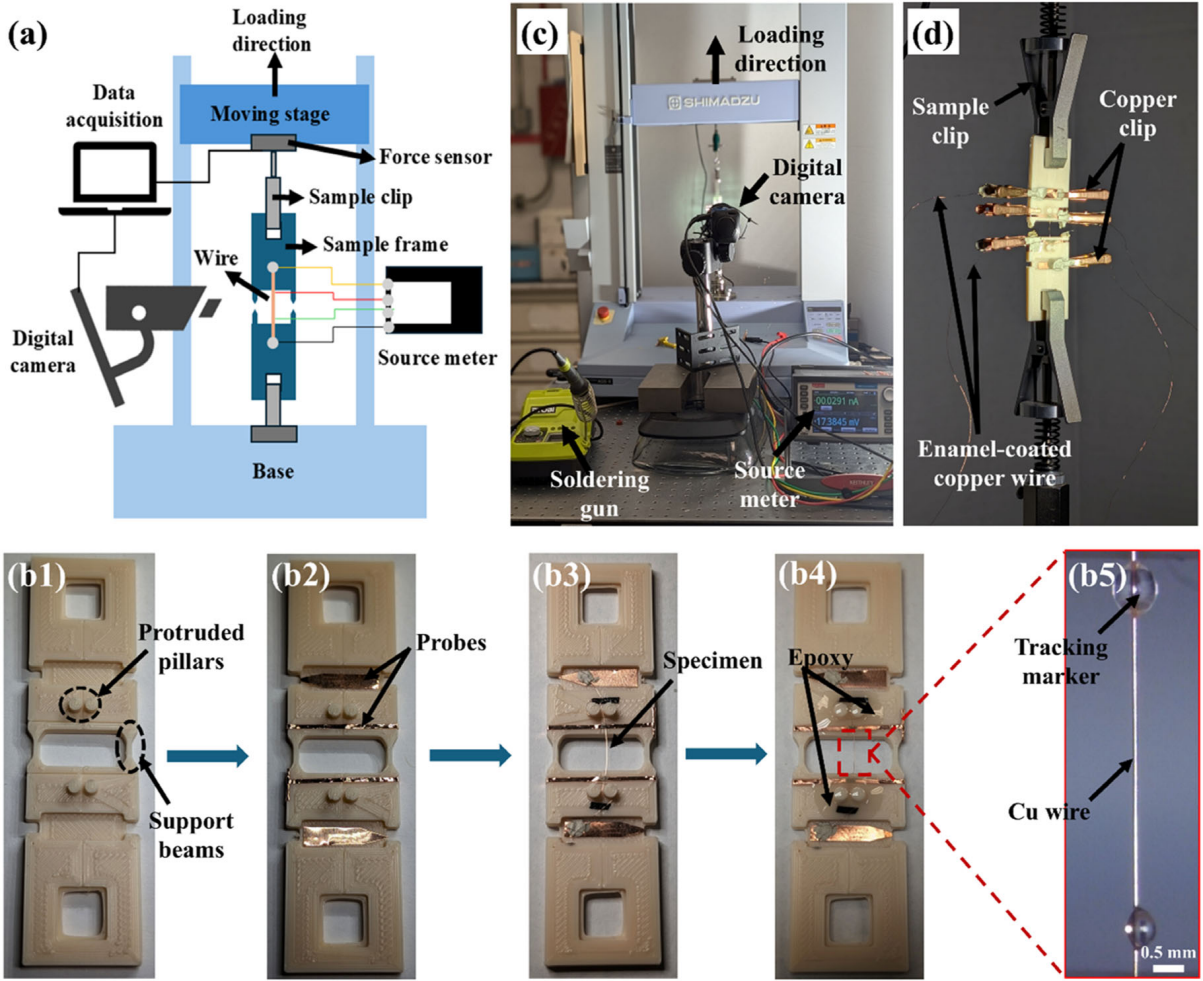
Electromechanical testing setup: a) schematic diagram illustrating the test configuration, including the wire specimen, moving stage, digital camera, and sourcemeter connections; b1–b5) sequential assembly steps of the 3D‐printed sample holder; c) a photograph of the actual experimental setup displaying the tensile stage, digital camera, and electrical measurement equipment; and d) detailed close‐up highlighting the copper clips and enamel‐coated copper wires connecting the four‐point probes to the sourcemeter.

An electromechanical tester shown in **Figure** **9**a consists of a commercial tensile tester, a customized 3D‐printed sample holder assembled with a wire specimen, a digital camera, a source meter, and a data acquisition system. Figure 9b1 shows the detailed design of a 3D‐printed sample holder, including two pairs of grooves to define the location and width of four electrical probes, two pairs of protruded pillars, two rectangular through holes, and two supporting beams. To mount a specimen onto a sample holder, we have developed the following protocols. First, thin copper foils were inserted into the four grooves with predefined dimensions as shown in Figure 9b2. This design ensured the accurate locations of the probes for four‐point electrical measurements and, as a result, allowed consistent electrical characterization by using an identical gauge length. Second, a wire specimen was carefully loaded onto the four probes in two steps (see Figure 9b3): 1) the wire passed through a small gap between each pair of the protruded pillars and, therefore, aligned in a loading direction, and 2) the specimen looped around pillars to mechanically anchor it and, as a result, prevented possible slips between the wire and holder. Third, conductive silver paste and epoxy glue were applied to achieve both robust electrical contacts and mechanical rigidity (see Figure 9b4). Finally, the wire specimen was decorated with two tracking markers in Figure 9b5 for measuring its deformation under uniaxial tension. The markers, created by applying a small amount of epoxy glue, were used as a reference for automated Digital Image Correlation (DIC).

After completion of the sample assembly, the holder was loaded onto the commercial uniaxial tester (AGS‐10kNXD, Shimadzu) by using the two sample clips shown in Figure 9d. The teeth of mechanical clips and the rectangular through holes had nearly identical dimensions to secure tight clamping. The four probes in the sample holder were connected to a source meter (2450 SourceMeter, Keithley) using four enamel‐coated copper wires for electrical measurements of the specimen concurrently with a mechanical tensile test (Figure 9c). During these sample handling steps, the sample holder prevented unwanted mechanical load on the specimen because the specimen was slightly curved, and its two ends were mechanically supported by the support beams (see Figure 9b).

The two support beams were thermally removed, just before performing the electromechanical test, by a soldering iron to separate the upper and lower parts of the sample holder. During this step, the resistance of the specimen was continuously monitored to avoid any unwanted plastic deformation, indicated by an unusual increase in resistance. A series of images was taken from the gauge length of the specimen using a digital camera (H800‐2713S‐3MF, Amscope) at 2 s intervals and then analyzed by a MATLAB‐based DIC algorithm to measure the distance between the two track markers. Using MATLAB code, a data acquisition system synchronized all data from the camera, source meter, and uniaxial tester and obtained the concurrently measured electrical and mechanical responses of each specimen. It is worth noting that Figure S12 and S13 in the Supporting Information underscore the importance of reliable experimental protocols by showing different types of experimental errors, for example, unstable electrical and/or mechanical measurements, due to slips between the specimen and holder. Our developed experimental approach enables analysis of electromechanical coupling behavior of the microscale graphene–copper composite wire and is adaptable to specimens with various dimensions, for example, wire, film, and porous body, by changing the design of the sample holder, providing a critical tool for future studies in graphene–metal composites.

##### Synthesis of Axially Continuous Graphene–Copper Wires

High‐purity copper wires with 80 μm diameter were purchased from California Fine Wires, California, USA. Graphene was grown on these wires using a CVD process where the focus was on the high‐quality and reproducible graphene coating on copper wires without SiO_*x*_ impurities.^[^ ^64^, ^65^ ^]^ Before CVD, the wires were carefully wound around a metal frame to avoid any material damage, for example, by excessive deformation, and then the frame was inserted into a furnace quartz tube for CVD graphene growth.

**Figure 10:**
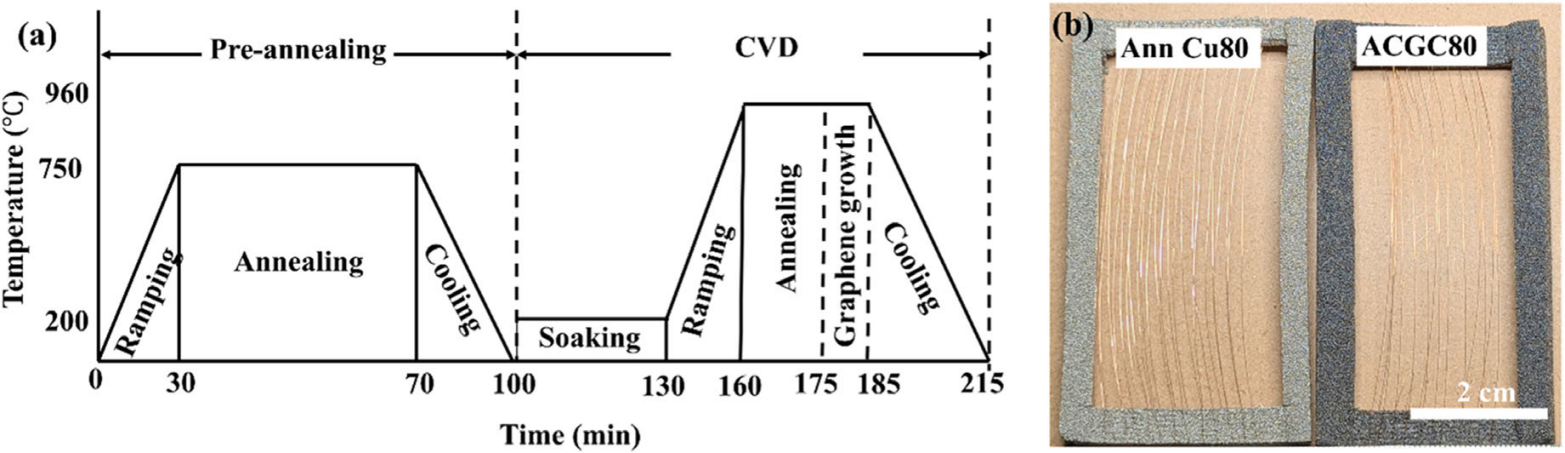
a) Detailed procedures to synthesize ACGC80, consisting of preannealing and CVD. b) Optical images of (left) annealed copper (Ann Cu80) and (right) axially continuous graphene–copper (ACGC80).

**Figure** **10**a shows the detailed recipe to synthesize ACGC80. Initially, a vacuum (<10^−2^ mbar) and purging with argon gas were performed thrice. Then, the copper wires underwent a preannealing step at 750 °C for 40 min in a reduction environment (1500 sccm of argon and 200 sccm of hydrogen flow) to eliminate any pre‐existing oxides. Hydrogen acts as a reducing agent by reacting with copper oxides, such as CuO or Cu_2_O, to form volatile compounds like H_2_O that can be easily removed.^[^ ^66^, ^67^, ^68^ ^]^ In addition, the elevated temperature helps break down oxides and smooths the copper surface.^[^ ^66^, ^67^, ^69^, ^70^, ^71^ ^]^ After preannealing, a soaking process was conducted at 200 °C for 30 min and a ramping step for another 30 min under an argon (760 sccm) and hydrogen (30 sccm) atmosphere. The furnace temperature was raised to 960 °C, and then the wires were annealed for 15 min at that temperature under an argon (1500 sccm) and hydrogen (100 sccm) atmosphere to remove possible residue on copper wires. Graphene growth was initiated by introducing a 10 sccm benzene flow for 10 min, during which the benzene decomposed into carbon atoms to form graphene on the copper wire. Finally, the samples were cooled down to room temperature. Annealed wires (hereafter Ann Cu80) were prepared under the identical conditions of ACGC80, with the only difference being the exclusion of the carbon precursor (i.e., benzene). Ann Cu80 samples were compared to ACGC80 samples to quantify Gr‐enhanced electromechanical properties. Figure 10b shows (left) the Ann Cu80 and (right) ACGC80 wires wrapped around the rectangular Ni frames after the completion of the sample preparation.

##### Characterization

Graphene quality, continuity, and layer number in ACGC80 and GCu foil specimens were analyzed via custom‐built Raman spectroscopy (532 nm, 6.0 mW). Microstructure was examined using OM (VHX‐7000, Keyence), SEM (Helios 5 UX, ThermoFisher), and SEM with EBSD (Auriga, Zeiss). Dislocation density in GCu and Ann Cu foils was estimated using XRD (Malvern PANalytical Aeris).

## Supporting Information

Supporting Information is available from the Wiley Online Library or from the author.

## Conflict of Interest

The authors declare no conflict of interest.

## Supporting information

Supplementary Material

## Data Availability

The data that support the findings of this study are available from the corresponding author upon reasonable request.
