# Supplementary material for: Electromechanical Behavior of Axially Continuous Graphene–Copper Wires
Source: Small Sci. 2025 Oct 14;5(12):e202500406. doi: 10.1002/smsc.202500406 (PMC12697877; doi:10.1002/smsc.202500406)
Supplement: Supplementary file 1 — Supplementary Material [file SMSC-5-e202500406-s001.pdf]

Supplementary Material of

Electromechanical Behavior of Axially Continuous Graphene–Copper (ACGC) Wires

Uschuas Dipta Das[1], Wonjune Choi[1,2], Hamid Safari[1], Jiali Yao[3], and Wonmo Kang[1,3,*]

[1]Mechaincal Engineering, School for Engineering of Matter, Transport and Energy, Arizona State University, Tempe, AZ 85281, USA

[2]Department of Materials Science and Engineering, Dankook University, Cheonan-si, Chungcheongnam-do 31116, Republic of Korea

[3]Materials Science and Engineering, Fulton Schools of Engineering, Arizona State University, Tempe, AZ 85287, USA

**Corresponding Author:* Wonmo.Kang@asu.edu

Thermo-electrical analysis of copper wire

A copper wire of length L and diameter d=2r, with both ends maintained at the ambient temperature of 298 K, can be considered to theoretically evaluate how the current density influences the Joule heating phenomenon (see Figure S1). When a specific current density, J (A/mm²), is applied to the copper wire, joule heating causes a volumetric heat generation. The generated thermal energy dissipates through conduction, convection, and radiation heat transfer. The rate of heat generation in the copper wire due to Joule heating is given by P = ρJ^2 $\bar{\nabla}$, where ρ denotes the resistivity of the material and $\bar{\nabla}$ represents the volume of the wire specimen. It is well established that conductive heat flux q_{ax} occurs axially through the cross-sectional area (A_{CS} = πr^2), while convective (q_{ro}) and radiative (q_R) heat flux occurs via the circumferential area (A_{CF} = $2\pi rL$) [1]. Consequently, the change in thermal energy can be expressed as:

$$\Delta U = \rho J^2 \bar{\nabla} - (2A_{CS}q_{ax} + A_{CF}q_{ro} + A_{CF}q_R) \qquad \text{Equation S1}$$

Equation S1 can be modified further to evaluate the change in the thermal energy per unit volume:

$$\frac{\Delta U}{\bar{\nabla}} = \rho J^2 - \frac{(2A_{CS}q_{ax} + A_{CF}q_{ro} + A_{CF}q_R)}{\bar{\nabla}} = \rho J^2 - \frac{1}{r}\left\{\frac{D}{L}q_{ax} + 2(q_{ro} + q_R)\right\} \qquad \text{Equation S2}$$

Equation S2 suggests that a lower applied current density and electrical resistivity, along with a higher heat dissipation rate, result in a reduced thermal energy change per unit volume in the specimen. The high thermal conductivity and low electrical resistivity of copper make it advantageous, leading to less

heat generation due to Joule heating and efficient heat dissipation. Small-scale copper wires, with their high aspect ratio, further enhance heat dissipation and reduce volumetric heat generation compared to bulk samples. Apparently, the radiative heat transfer can be neglected since the temperature difference between the ambient temperature and the temperature of the wire is very small. Finite Element Analysis (FEA) is performed using COMSOL to predict the time and position-dependent temperature profile for various current densities, considering an 80-μm-diameter as-received copper (AR Cu80) wire with a length of 10 mm. Considering free convection, the convection heat transfer coefficient was approximated as 10 Wm^{-2} K^{-1} [2]. The material properties of copper considered in the COMSOL simulation can be found in Table S1.

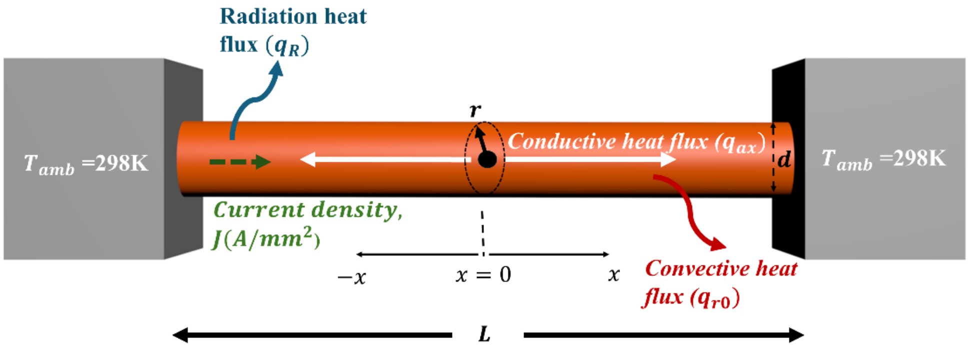

Figure S1. Schematic of an AR Cu80 wire specimen with an applied current and different heat transfer modes.

The data obtained from the thermo-electrical analysis of the AR Cu80 wire was depicted in Figure S2(a), which clearly displays the temperature distribution along the axial direction of the specimen at the current density of 4, 50, 100, 200, and 300 A/mm². Since both ends of the wire were considered at the constant ambient temperature, the maximum temperature of the specimen was recorded at the midpoint. Figure S2(b) further illustrates the time-dependent evolution of the average specimen temperature at different current densities. As the current density increases, a pronounced rise in temperature becomes evident, indicating greater heat generation in the specimen at higher current densities. The most significant temperature increase (~45 °C) was observed at 300 A/mm², while at 50 A/mm², the temperature rise remained below 1.5 °C, indicating that the wire's electrical resistivity would be relatively stable under moderate current flow. Given the temperature coefficient of resistance for copper (~0.39% per °C), even small temperature changes can substantially influence resistance measurements during electromechanical characterization. Consequently, a lower current density of approximately 4 A/mm² was selected for subsequent experiments to ensure that any temperature rise in the microscale wire specimen would be negligible.

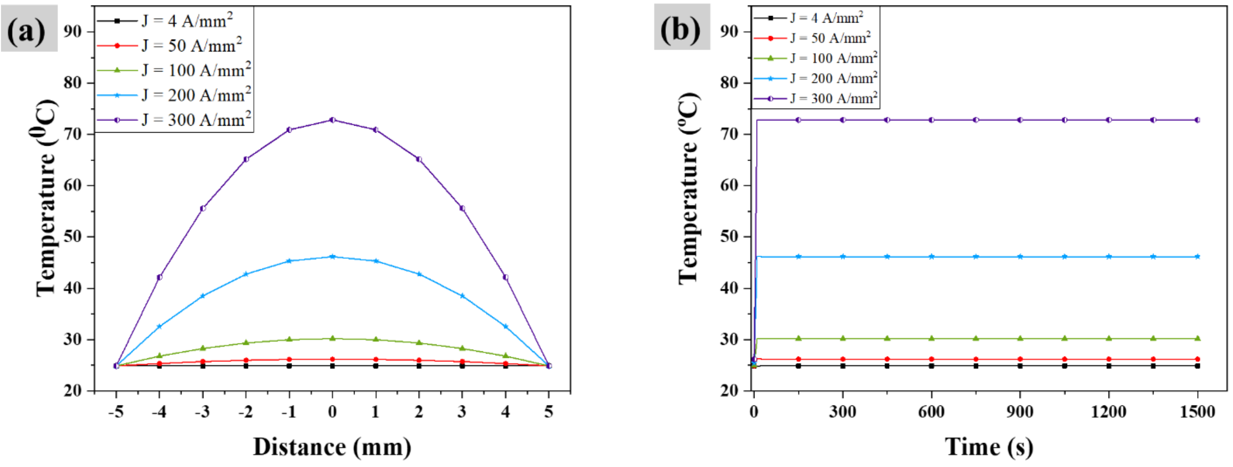

Figure S2. Thermo-electrical analysis of an 80-µm-diameter as-received copper wire: (a) Simulated temperature profiles along the wire length at different current densities, and (b) Time-dependent evolution of average wire temperature.

Table S1. Material properties of Copper (Cu) in simulation.

**Material Properties**	**Value**
Electrical conductivity [S/m]	5.998×10^{7}
Coefficient of thermal expansion [1/K]	17×10^{-6}
Heat capacity at constant pressure [J/(kg.K)]	385
Density [kg/m³]	8960
Thermal conductivity [W/(m.K)]	400
Young's modulus [GPa]	110
Poisson's ratio	0.35
Reference resistivity [Ω·m]	1.72×10^{-8}
Resistivity temperature coefficient [1/K]	39×10^{-4}

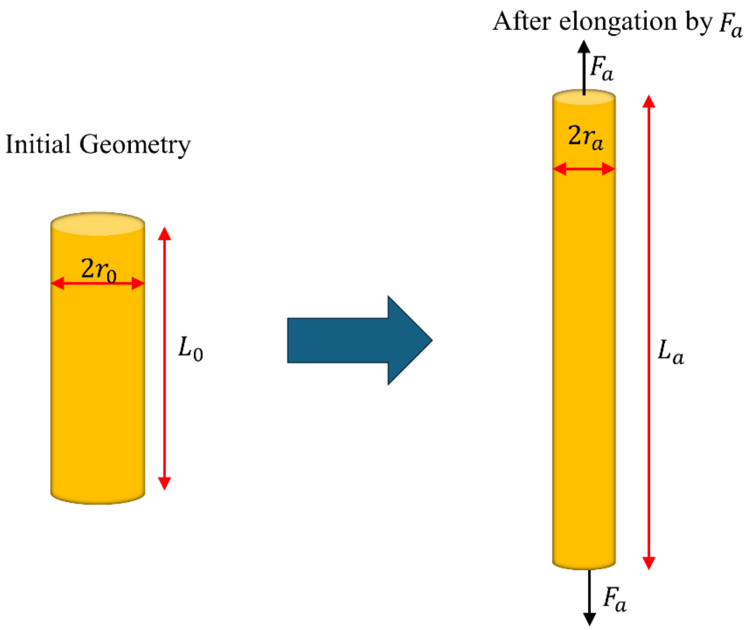

Figure S3. Schematics of a wire specimen before and after applying tensile load F_a.

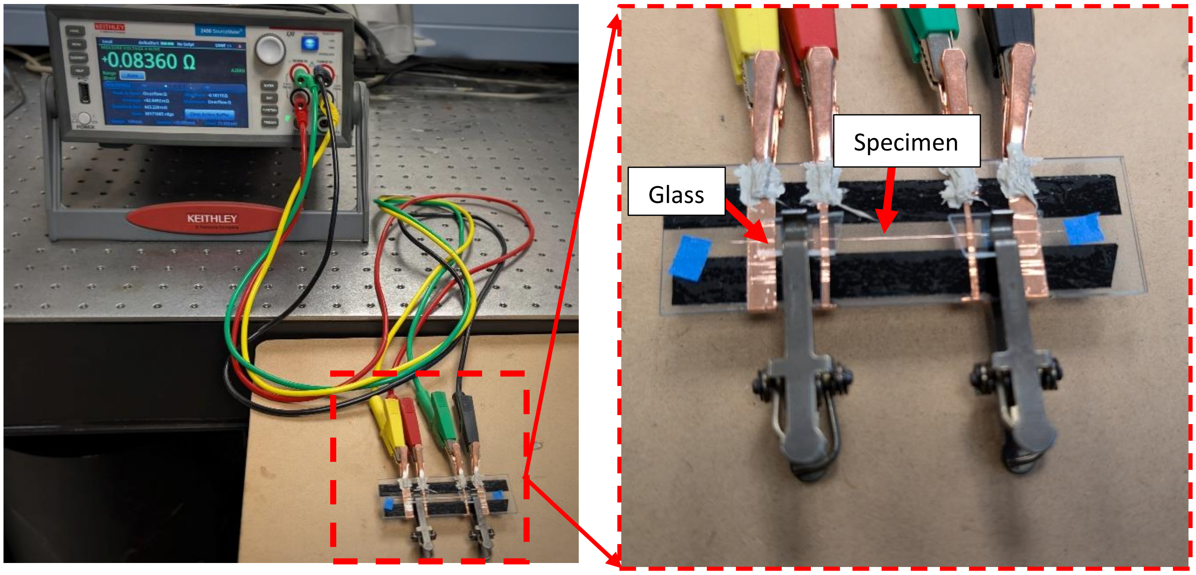

Figure S4. Our custom-made four-point probe apparatus to measure the resistivity of wire specimens. Two small glass slides and metal clips were used to make proper and consistent contact between the probes and the specimen.

Table S2. Comparison between the resistivity measured of AR Cu80 specimens by the four-point probe resistivity measurement setup and the (initial) resistivity measured in the electromechanical tester.

Measurement Technique	Sample ID	Resistivity ($\times 10^{-8}\Omega.m$)	Standard Deviations ($\times 10^{-11}\Omega.m$)	Mean Resistivity ($\times 10^{-8}\Omega.m$)
Four-Point Probe	1	1.757	2.147	1.760
	2	1.763		
	3	1.759		
	4	1.760		
Electromechanical Test (initial resistivity)	1	1.770	1.615	1.770
	2	1.769		
	3	1.772		

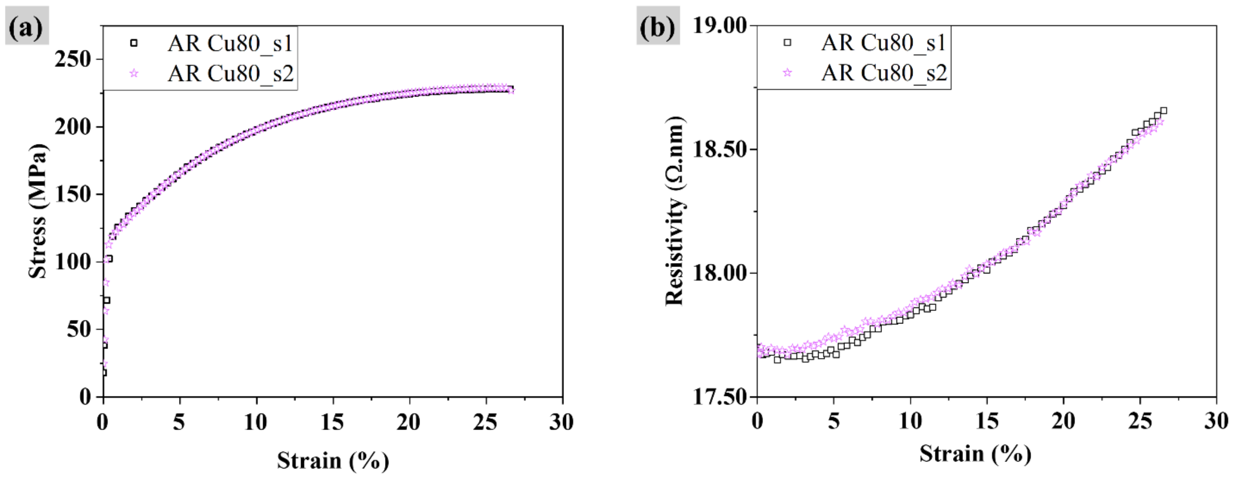

Figure S5. Electromechanical characterization of AR Cu80 wire specimens: (a) stress-strain curve and (b) resistivity as a function of applied strain.

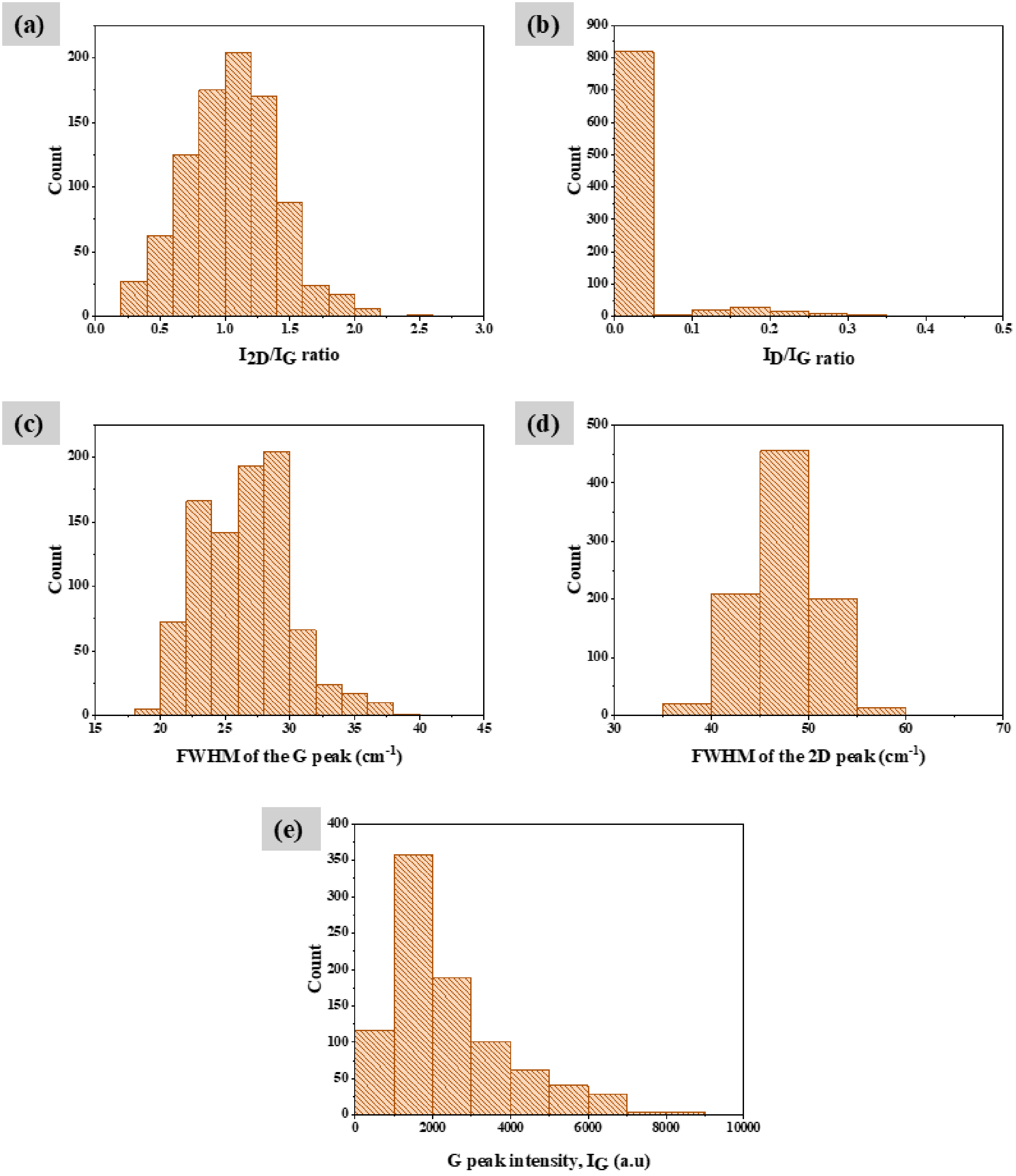

Figure S6. The histograms of graphene-coated copper composite (ACGC80) wire show the distribution of the (a) I_{2D}/I_G ratio, (b) I_D/I_G ratio, (c) FWHM of G peak, (d) FWHM of 2D peak, and (e) intensity of G peak.

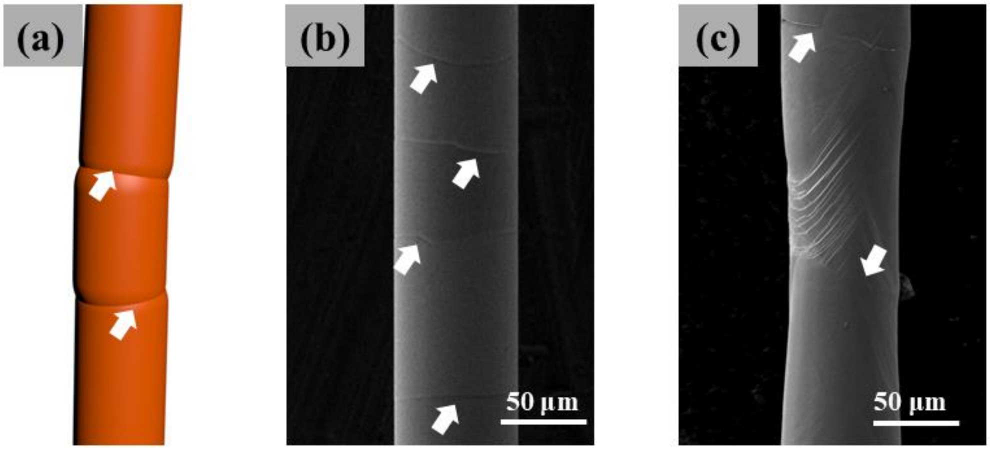

Figure S7. a) Schematic of the bamboo-like microstructure forms in ACGC80 and Ann Cu80 wires due to thermal annealing. The bamboo-like microstructure is evident in the SEM images of 80 µm-diameter ACGC wire (b) as prepared and (c) after fracture. White arrows were used to indicate grain boundaries.

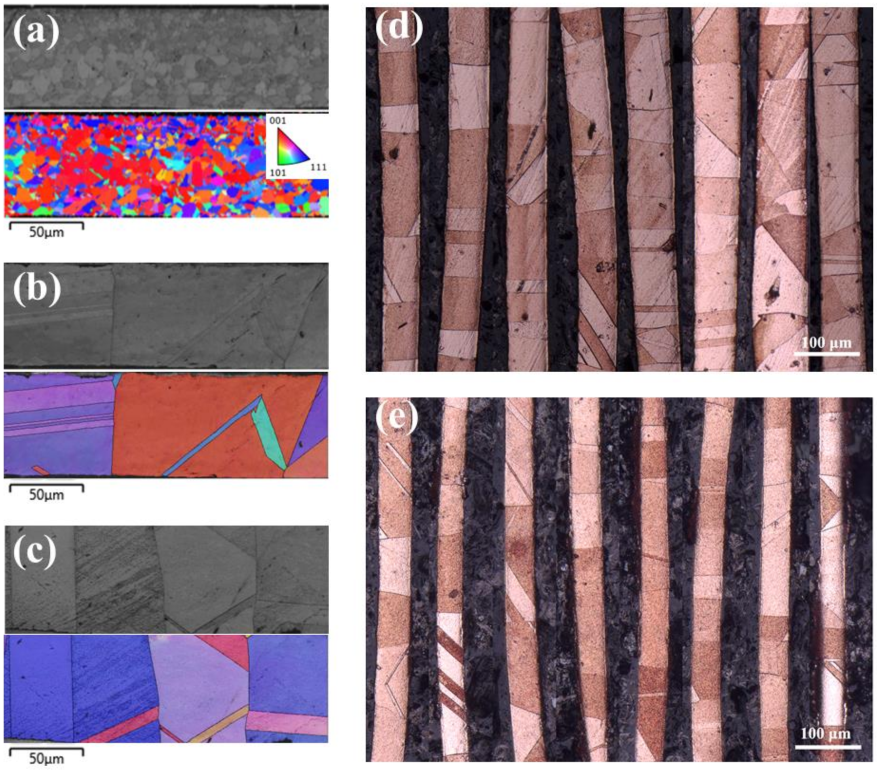

Figure S8. Electron backscatter diffraction (EBSD) analysis of (a) AR Cu80, (b) Ann Cu80, and (c) ACGC80 with band contrast images (top) and corresponding inverse pole figure (IPF) maps (bottom). Optical microscopy (OM) image of mechanically polished and then chemically etched (d) Ann Cu80 and (e) ACGC80 samples used for grain size estimation. Both EBSD and OM images reveal a significant grain size increasing from a few microns to tens of microns and the formation of a similar bamboo-like microstructure in Ann Cu80 and ACGC80 as a result of thermal annealing.

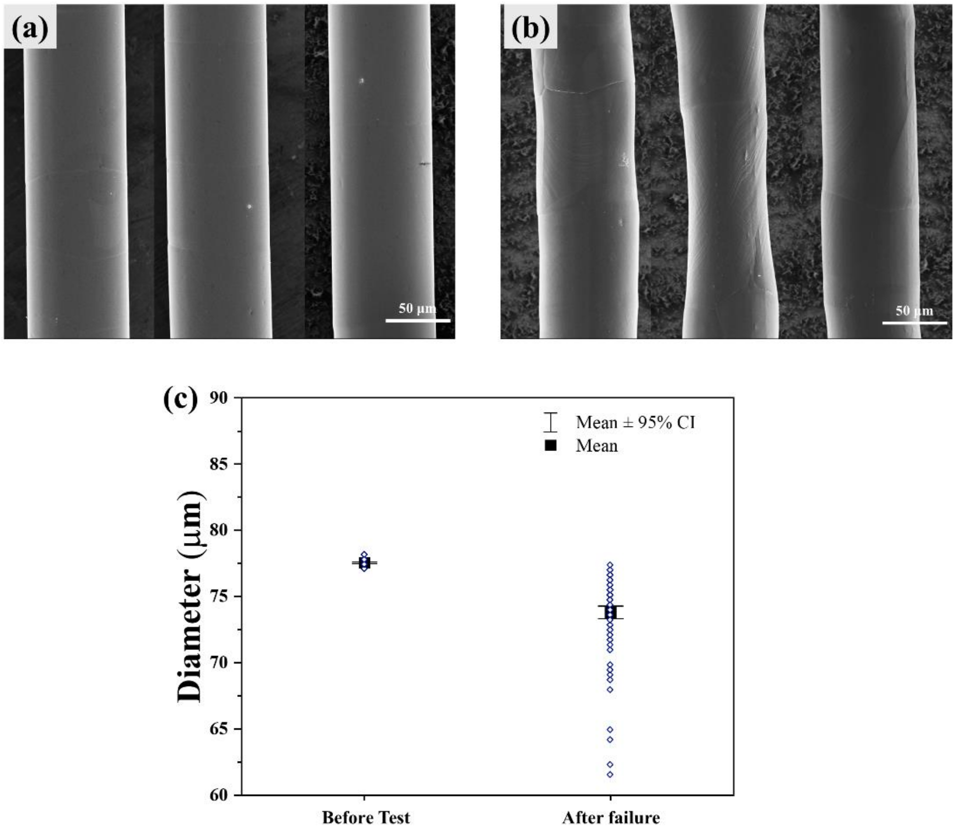

Figure S9. SEM micrographs of Ann Cu80 wires reveal (a) uniform cross-sections before testing and (b) localized deformation behavior after failure. (c) Diameter measurements taken at multiple locations before and after testing demonstrate a wider distribution after failure, confirming the localized deformation behavior in Ann Cu80, consistent with ACGC80 wires.

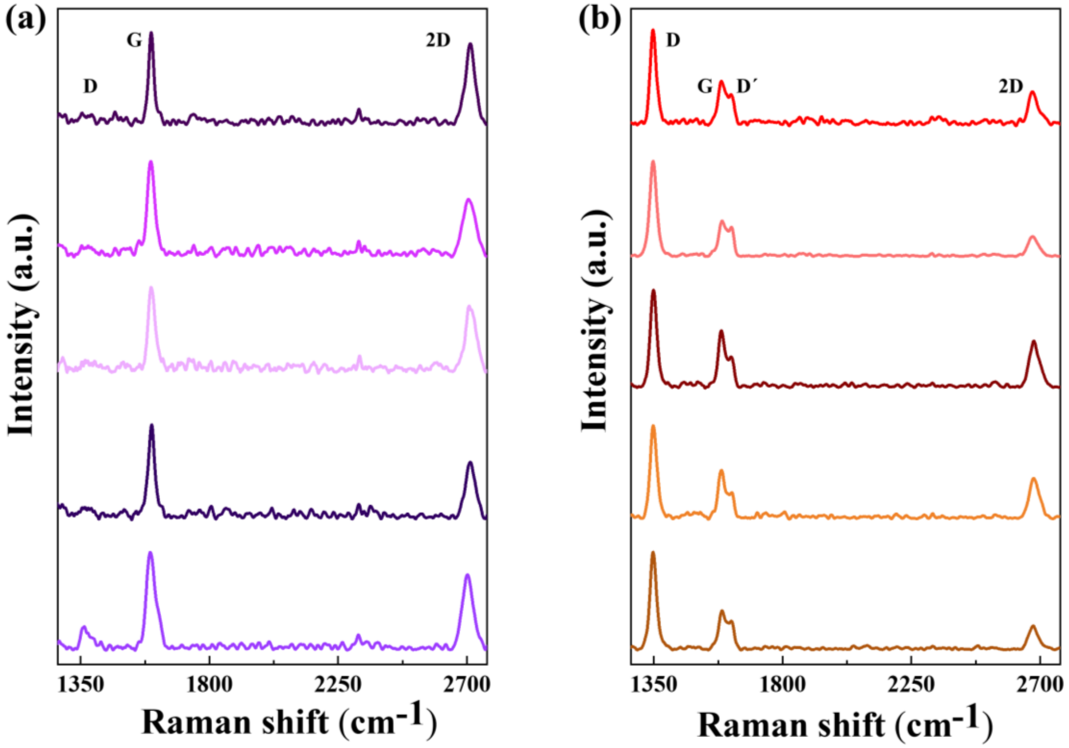

Figure S10. Raman spectroscopy of graphene-coated foil (a) before rolling and (b) after rolling process. Five points were randomly chosen in both types of specimens, and a significant increase in D peak and the emergence of a pronounced D' peak were observed after the rolling process, suggesting significant disorder and defects in graphene.

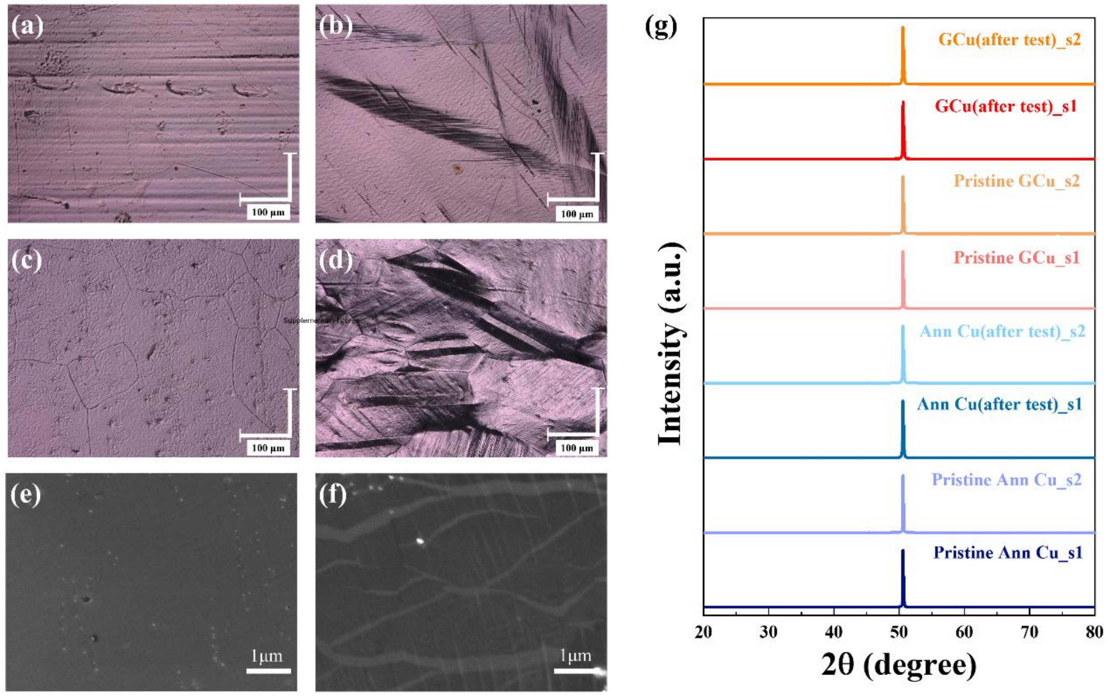

Figure S11. Optical microscopy (OM) images of (a) pristine annealed copper (Ann Cu) foil, (b) Ann Cu foil after fracture, (c) pristine graphene-coated copper (GCu) foil, and (d) GCu foil after fracture. SEM image of (e) fractured Ann Cu foil, (f) fractured GCu foil, highlighting clear morphological differences, with a pronounced crack visible in the GCu foil—absent in the Ann Cu sample. X-ray diffraction (XRD) patterns of the corresponding samples indicate a dominant Cu(200) crystallographic texture in both annealed and graphene-coated specimens.

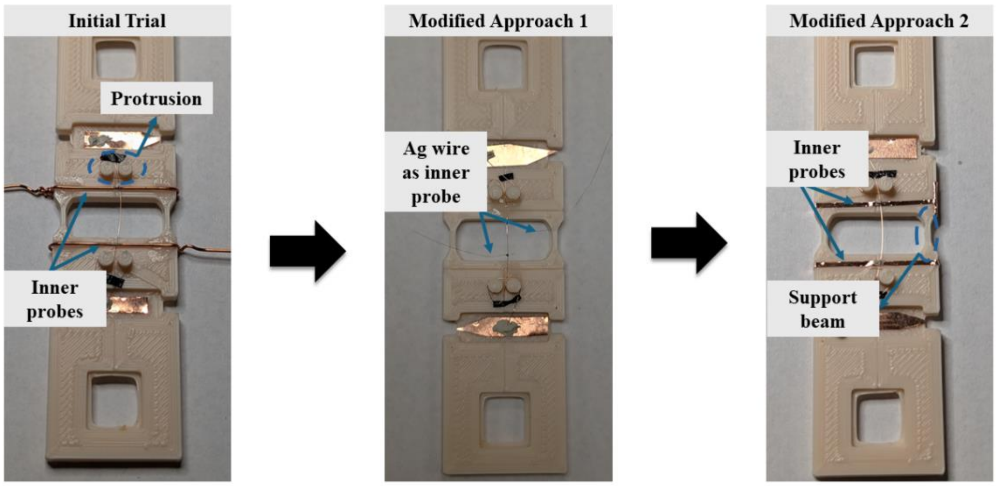

Figure S12. Image of the sample holder designs explored during the trial-and-error process to identify the most suitable configuration for electromechanical characterization of microscale wires.

The sample holder configuration used in this study was developed through several trials and iterative modifications, successfully capturing both mechanical and electrical responses simultaneously. In the initial

trials, 250-µm-diameter copper wires (first approach) and flexible silver wire knots (second trial) were used as inner probes (see Figure S12). However, both approaches suffered from significant issues like sudden stress drop and electrical contact issues due to slippage, preventing further use of these approaches (see Figure S13(a) and (b)). Finally, the sample frame was modified utilizing copper tape as the inner probes were mounted on the sample frame. The reason for using copper tape as the inner elctro probe is that it is easy to mount on top of the sample holder, and the thin flat tape allowed the epoxy to fill all voids, ensuring that the wire remained stationary within the epoxy-covered region while preventing any unwanted slip between the inner probes and the specimen during the tensile test (see Figure S13(c)).

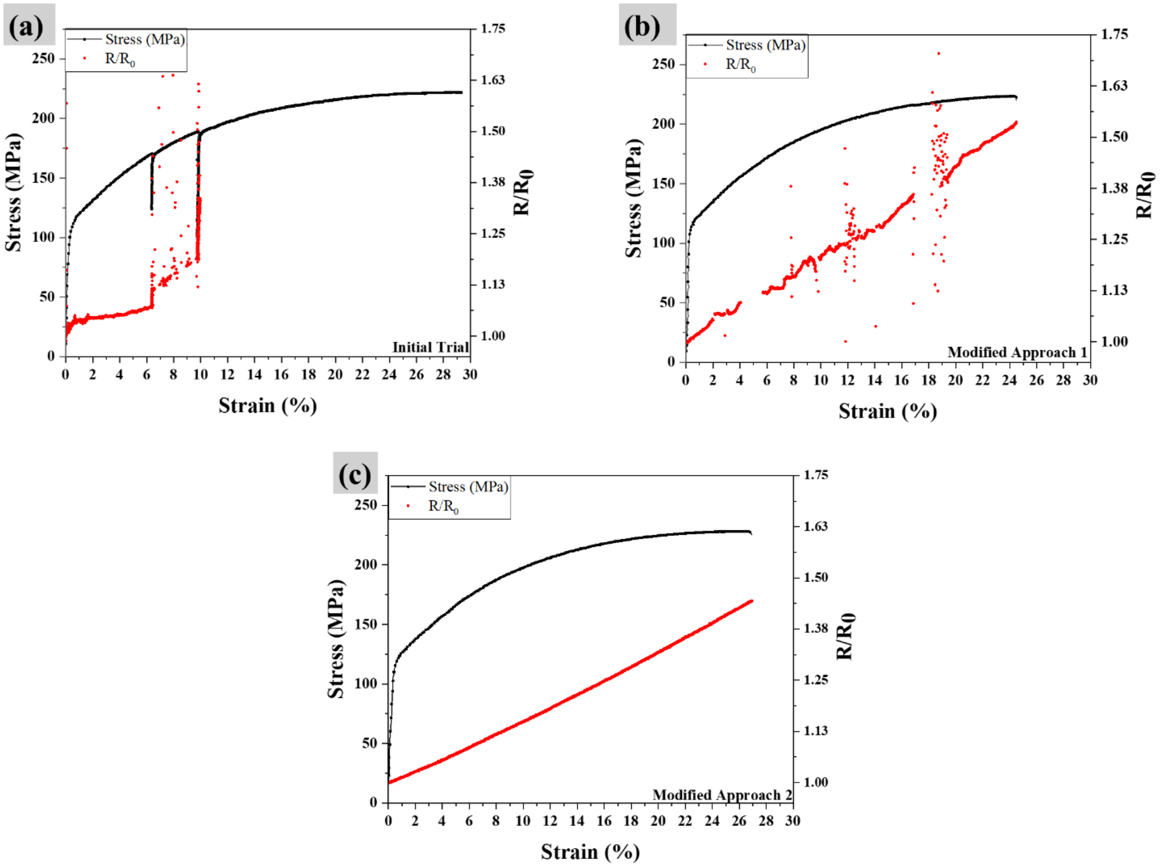

Figure S13. Electro-mechanical characterization of 80µm as-received copper wires (AR Cu80) using various approaches: (a) Initial trial employing copper wire as inner probes, (b) Modified approach 1 utilizing flexible silver wire as inner probes, and (c) Modified approach 2 incorporating copper tape as inner probes. In all methods, the specimen was connected to the electrodes using a small amount of silver paste, followed by epoxy application after curing.

REFERENCES

1. Choi, W., et al., *Electro-thermo-mechanical characterization of microscale Ti-6Al-4V wires using an innovative experimental method.* Materials Characterization, 2022. **188**: p. 111927.
2. Kosky, P., et al., *Chapter 14 - Mechanical Engineering*, in *Exploring Engineering*, R.B. Philip Kosky, William Keat, George Wise, and E.E.F. Edition), Editors. 2013, Academic Press. p. 317-340.
